# Early identification of sepsis-induced acute kidney injury utilizing NGAL, Cystatin C, urine output, and serum creatinine in a porcine ( *Sus scrofa*) model of septic shock: A comparative study of fluid and vasopressor strategies

**DOI:** 10.12688/f1000research.182793.1

**Published:** 2026-07-06

**Authors:** Andriamuri Primaputra Lubis, Syah Mirsya Warli, Tasrif Hamdi, Gunanti Gunanti, Antonius Hocky Pudjiadi, Muhammad Rusda, Cut Meliza Zainumi, Raka Jati Prasetya

**Affiliations:** 1 Philosophy Doctor in Medicine Program, Faculty of Medicine, Universitas Sumatera Utara, Medan, North Sumatra, Indonesia; 2 Department of Anesthesiology and Intensive Care, Faculty of Medicine, Universitas Sumatera Utara Fakultas Kedokteran, Medan, North Sumatra, Indonesia; 3 Division of Urology, Department of Surgery, Universitas Sumatera Utara Fakultas Kedokteran, Medan, North Sumatra, Indonesia; 4 Division of Surgery and Radiology, School of Veterinary Medicine and Biomedical Sciences, Institut Pertanian Bogor, Bogor, West Java, Indonesia; 5 Department of Child Health, Faculty of Medicine, Universitas Indonesia, Cipto Mangunkusumo Hospital, Jakarta, Indonesia, Universitas Indonesia, Depok, West Java, Indonesia; 6 Department of Obstetrics and Gynecology, Universitas Sumatera Utara Fakultas Kedokteran, Medan, North Sumatra, Indonesia

**Keywords:** Sepsis; Acute Kidney Injury; Neutrophil Gelatinase-Associated Lipocalin; Cystatin C; Norepinephrine

## Abstract

**Background:**

Sepsis-associated acute kidney injury (SA-AKI) is a common complication of septic shock and is associated with prolonged ICU stay and increased mortality. Early detection of renal injury remains challenging because conventional markers such as serum creatinine and urine output often reflect delayed functional changes. Structural biomarkers, including neutrophil gelatinase-associated lipocalin (NGAL) and cystatin C, have emerged as promising indicators for the earlier detection of kidney injury. The aim of the study was to compare early structural biomarkers (NGAL and cystatin C) with conventional functional markers (serum creatinine and urine output) in a porcine model of septic shock, and to evaluate their temporal patterns under three different fluid and/or vasopressor resuscitation strategies.

**Methods:**

A randomized controlled experimental study was conducted using six *Sus scrofa* pigs subjected to standardized septic shock induction. Animals were allocated into three groups: fluid resuscitation with Ringer’s lactate (30 mL/kg), fluid combined with early norepinephrine infusion, and early norepinephrine with maintenance fluids. Plasma NGAL, cystatin C, serum creatinine, and ultrasonographically assessed urine dynamics were measured sequentially. Hemodynamic parameters were continuously monitored. Data were analyzed using repeated-measures and nonparametric tests with a significance level of p < 0.05.

**Results:**

NGAL and cystatin C demonstrated earlier temporal changes compared with serum creatinine across all groups. Ringer’s lactate only and Ringer’s lactate-norepinephrine groups showed declining biomarker trends after intervention, whereas the early norepinephrine group exhibited delayed increases. Serum creatinine remained stable during the observation period. Intergroup differences were not statistically significant.

**Conclusion:**

Structural biomarkers exhibited earlier dynamic changes than serum creatinine in this porcine septic shock model. Although statistically non-significant, distinct biomarker patterns were observed among resuscitation strategies, suggesting potential value of integrating sensitive renal biomarkers with individualized hemodynamic management for early detection of SA-AKI.

## 1. Introduction

Sepsis is a critical organ dysfunction resulting from an aberrant host response to infection and continues to be a predominant cause of morbidity and mortality globally. ^1^ Sepsis-associated acute kidney injury (SA-AKI) is one of the most prevalent and severe complications of septic shock, independently linked to extended intensive care unit (ICU) stay, progression to chronic kidney disease, and heightened mortality rates. ^2^ ^,^ ^3^ Despite advancements in sepsis management, the prompt identification of renal injury during the initial resuscitation phase poses a significant clinical challenge.

The current diagnostic criteria for acute kidney injury predominantly rely on functional markers, notably serum creatinine levels and urine output. ^4^ However, serum creatinine serves as a delayed and insensitive indicator of acute changes in glomerular filtration, particularly in critically ill patients undergoing fluid loading, haemodilution, altered muscle metabolism, and systemic inflammation. Neurohormonal responses, hemodynamic therapies, and fluid balance may all impact urine output, making it less reliable as a standalone sign of kidney damage. As a result, considerable structural damage to the kidneys may occur before traditional criteria for Acute Kidney Injury (AKI) are met.

Structural biomarkers of renal injury have emerged as promising instruments for the early detection of Acute Kidney Injury (AKI). ^4^ Neutrophil gelatinase–associated lipocalin (NGAL) is swiftly upregulated and released by damaged renal tubular epithelial cells following ischemic or inflammatory insults. ^4^ Cystatin C, a low–molecular weight protease inhibitor that is freely filtered by the glomerulus, offers a more stable indication of glomerular filtration independent of muscle mass and volume status. ^2^ ^,^ ^3^ Collectively, these biomarkers may facilitate earlier detention of renal injury prior to the manifestation of significant functional decline.

Fluid resuscitation and vasopressor treatment are the primary strategies for hemodynamic care in septic shock. ^3^ ^,^ ^4^ Current recommendations recommend the immediate administration of intravenous crystalloids, followed by vasopressors, to maintain adequate mean arterial pressure. However, aggressive fluid resuscitation may exacerbate endothelial dysfunction, interstitial edema, and renal congestion, ultimately worsening kidney damage. Conversely, the immediate infusion of norepinephrine may restore vascular tone, improve organ perfusion, and reduce excessive fluid exposure. The renal consequences of diverse fluid–vasopressor strategies during the first phase of septic shock, particularly with early kidney damage indicators, remain little defined.

Experimental animal models provide a controlled environment to investigate the pathophysiological mechanisms of SA-AKI and evaluate renal biomarkers under standardized hemodynamic conditions. The porcine ( *Sus scrofa*) model is especially suitable due to its significant anatomical, physiological, and hemodynamic resemblances to humans. We hypothesized that NGAL and cystatin C would detect sepsis-induced acute kidney injury earlier and with greater sensitivity than conventional renal parameters following septic shock and resuscitation. Therefore, the aim of this study was to compare early structural biomarkers (NGAL and cystatin C) with conventional functional markers (serum creatinine and urine output) in a porcine model of septic shock, and to evaluate their temporal patterns under three different fluid and vasopressor resuscitation strategies.

## 2. Methods

### 2.1 Study design

This study was a randomized, controlled experiment using a porcine model of septic shock. ^5^ All procedures were approved by the Institutional Animal Care and Use Committee and and adhered to national regulations for laboratory animal research, including the 3Rs (Reduction, Replacement, Refinement) and 5Fs (Freedom from Hunger and Thirst, Freedom from Discomfort, Freedom from Pain, Injury or Disease, Freedom to Express Normal Behavior, Freedom from Fear and Distress). ^6^ The experimental unit was an individual animal (one pig). Six animals were included in the study and randomly allocated to three treatment groups (n = 2 animals per group).

### 2.2 Data collection

Measures were implemented to mitigate animal suffering and decrease the number of animals utilized. In this study, the experimental animal model used consisted of male pigs ( *Sus scrofa*) aged fourteen to eighteen weeks, weighing between 55 and 60 kg. Before the trial, six healthy The sample size used in this study was based on the resource equation method. The study consisted of one control group and three treatment groups, with three repeated measurements performed at different time points: before sepsis induction, during sepsis/septic shock, and after therapy.

Sample size was determined prior to the experiment by considering the Reduction principle in large-animal research and applying the resource equation method. As this was an exploratory preclinical/translational study, the number of animals was kept to the minimum necessary while maintaining the ability to evaluate temporal changes in renal biomarkers and hemodynamic parameters in a septic shock model.

Six male pigs (Sus scrofa) were used as experimental units and were equally allocated to three resuscitation strategy groups (n = 2 per group): (1) a 30 mL/kg lactated Ringer’s group, (2) a 30 mL/kg lactated Ringer’s plus early norepinephrine group, and (3) an early norepinephrine group receiving maintenance lactated Ringer’s at 3 mL/kg/h. The analyzed sample size (n) for each outcome is reported in the Results tables. No animals, experimental units, or data points were excluded from the final analysis, provided that all measurements at T1, T2, and T3 were available.

Sus scrofa pigs of similar age and body weight were acclimatized. The animals were not allowed to eat for a night, but they may drink as much water as they desired. Each experimental animal was anesthetized with atropine sulfate 0.04 mg/kg body weight intramuscularly, ketamine HCl 10% at a dose of 20 mg/kg body weight intramuscularly, and xylazine HCl 2% at a dose of 2 mg/kg body weight intramuscularly. Anesthesia was maintained with propofol at a dose of 2–4 mg/kg body weight intravenously, supported by mechanical ventilation using a volume-controlled mode adjusted according to arterial blood gas analysis. Normal saline 0.9% at a dose of 3 mL/kg/hour was administered for maintenance fluid therapy.

After endotracheal intubation, the participants were mechanically ventilated with oxygen-air mixtures to keep the levels of oxygen and carbon dioxide in the blood normal. During the whole trial, heart rate, blood pressure, oxygen saturation, and temperature were all watched all the time. One hour after stabilization, hemodynamic parameters, which also served as indicators of volume adequacy, and blood samples were obtained for baseline measurements. This procedure was performed through the right femoral artery using an aseptic technique, and a thermistor-tipped transpulmonary thermodilution catheter (PiCCO, PULSION Medical Systems SE, Munich, Germany) was inserted into the right femoral artery for hemodynamic monitoring and temperature measurement.

Data collection and measurements were performed at three predefined time points: T1, during baseline data acquisition after the stabilization period; T2, at the time of sepsis induction/septic shock; and T3, after the post-resuscitation intervention. Hemodynamic parameters, laboratory examinations, and renal function assessments were evaluated at each time point to determine the physiological changes occurring throughout the experimental protocol.

Sepsis was induced in the experimental animals by administration of *Escherichia coli* endotoxin (lipopolysaccharide 0111:B4, Difco Laboratories, Detroit, MI) via continuous infusion initiated at a rate of 0.30 μg/kg/hour. Septic shock was considered established when evidence of organ dysfunction was present, indicated by a mean arterial pressure (MAP) ≤ 65 mmHg sustained for 30 minutes. ^6^

There were three treatment groups, and animals were randomly put into one of them. The Fluid Resuscitation Group: Animals received Ringer’s lactate at a dose of 30 mL/kg administered over 30 to 60 minutes, in accordance with contemporary sepsis resuscitation guidelines. ^7^ The Fluid plus Early Norepinephrine Group **:** Animals received Ringer’s lactate at a dose of 30 mL/kg along with a quick start to norepinephrine infusion, which started at 0.1 μg/kg/min and was changed to keep the mean arterial pressure at least 65 mmHg. If the MAP had not reached ≥65 mmHg, a 500 mL fluid bolus was administered in addition to norepinephrine (NE) at 0.2 μg/kg/minute. If the MAP remained <65 mmHg, the norepinephrine dose was further titrated up to 0.3 μg/kg/minute at each hemodynamic assessment until the target MAP ≥65 mmHg was achieved. Failure to achieve the target MAP despite these interventions was considered treatment failure. ^7^ The Early Norepinephrine with Maintenance Fluid Group **:** Animals received norepinephrine starting at 0.1 μg/kg/min from the beginning of septic shock, along with maintenance Ringer’s lactate infusion at 3 mL/kg/h. This was an early vasopressor approach meant to reduce fluid overload. ^7^ ^,^ ^8^ Hemodynamic targets were maintained throughout the observation period with regular adjustments of fluid administration and vasopressor dose.

Following a 10-day quarantine and acclimatization period, animals that were deemed healthy and met all study eligibility criteria were assigned sequential individual identification codes (P01–P06). Allocation to the study groups was performed using computer-based randomization with the RANDOM.ORG List Randomizer. The randomization list consisted of six allocation labels in a 1:1:1 ratio: two labels for the lactated Ringer’s group, two labels for the lactated Ringer’s plus early norepinephrine group, and two labels for the early norepinephrine with maintenance fluid group.

The allocation sequence was generated before the experiment by an independent staff member who was not involved in administering the interventions, measuring outcomes, performing biomarker assessments, conducting ultrasonographic examinations, or carrying out statistical analyses. To maintain allocation concealment, the randomization list was stored separately in a secure digital file accessible only to the staff member responsible for group allocation. Group assignments were revealed only after the animals had completed the acclimatization period, met all eligibility criteria, and undergone baseline measurements.

Potential confounding factors were minimized by using animals of the same sex with similar ages and body weight ranges; housing all animals in the same facility under individual housing conditions; applying identical quarantine and acclimatization procedures; and standardizing anesthesia, mechanical ventilation, septic shock induction, and hemodynamic monitoring protocols. Outcome measurements were performed at the predefined time points (T1, T2, and T3) in accordance with the study protocol.

Blood samples were collected at predetermined intervals to assess plasma NGAL, cystatin C, and serum creatinine, which are frequently employed biomarkers for the evaluation of sepsis-associated acute kidney injury. NGAL and cystatin C biomarker concentrations were measured using the enzyme-linked immunosorbent assay (ELISA) method with a particle-enhanced turbidimetric immunoassay technique, in accordance with the manufacturers’ instructions. All samples were handled and assessed in a blind manner.

Urine output was indirectly measured using consecutive ultrasonographic assessments of urinary bladder volume. ^9^ ^,^ ^10^ Changes in bladder distension over time were used to show how urine output changed during septic shock. No standard geometric formulas were employed, and urine production was neither computed nor quantitatively reported in milliliters per kilogram per hour.

Blood samples were collected at predetermined intervals to assess plasma neutrophil gelatinase-associated lipocalin (NGAL), cystatin C, and serum creatinine as biomarkers of sepsis-associated acute kidney injury. Plasma NGAL concentrations were measured using a Porcine NGAL ELISA Kit (Fine Test, Wuhan Fine Biotech Co., Ltd., Wuhan, China; Catalog No. EP0121), while plasma cystatin C concentrations were measured using a Porcine Cystatin C (Cys-C) ELISA Kit (Fine Test, Wuhan Fine Biotech Co., Ltd., Wuhan, China; Catalog No. EP0046). Both biomarkers were quantified using a sandwich enzyme-linked immunosorbent assay (ELISA) technique according to the manufacturer’s instructions. All samples were processed under identical laboratory conditions and analyzed in a blinded manner to minimize measurement bias. Biomarker analyses were performed using the same lot of assay kits to reduce inter-assay variability.

Sepsis was induced by continuous intravenous infusion of *Escherichia coli* endotoxin (lipopolysaccharide O111:B4; Difco Laboratories, Detroit, MI, USA). Animals received Ringer’s lactate solution for fluid resuscitation and norepinephrine infusion for hemodynamic support according to group allocation. Anesthesia was induced using atropine sulfate (0.04 mg/kg intramuscularly), ketamine hydrochloride 10% (20 mg/kg intramuscularly), and xylazine hydrochloride 2% (2 mg/kg intramuscularly), and maintained with intravenous propofol (2–4 mg/kg). Mechanical ventilation was provided throughout the experimental period. At the completion of the experiment, all animals were humanely euthanized under deep general anesthesia. Anesthesia was maintained with continuous intravenous infusion of propofol (6–10 mg/kg/h) and fentanyl (5–25 μg/kg/h). Adequate anesthetic depth was confirmed before euthanasia by the absence of spontaneous movement, loss of jaw tone and corneal reflexes, and stable respiratory and hemodynamic parameters.

Euthanasia was performed by slow intravenous administration of magnesium sulfate (MgSO _4_) 50% injection (500 mg/mL, Magnesium Sulfate Heptahydrate 50% w/v Injection, Pfizer/Hospira, Lake Forest, IL, USA) at a dose of 200–250 mg/kg body weight until cardiac arrest occurred. Mechanical ventilation and physiologic monitoring were maintained throughout the procedure. Death was confirmed by the absence of pulse, heart sounds, respiratory sounds, spontaneous respiration, corneal reflexes, and response to painful stimuli. Monitoring was continued until cardiac and respiratory arrest were sustained. If any signs of life persisted, additional euthanasia measures were performed under veterinary supervision until death was confirmed.

All euthanasia procedures were performed by or under the supervision of a licensed veterinarian and were conducted in accordance with the AVMA Guidelines for the Euthanasia of Animals: 2020 Edition. Consistent with these guidelines, magnesium sulfate was administered only to animals that were unconscious and maintained under deep general anesthesia. Inclusion and exclusion criteria were established before the study commenced. Eligible animals were clinically healthy male pigs ( *Sus scrofa*), aged 14–18 weeks, weighing 55–60 kg, with relatively homogeneous age and body weight ranges, and having completed a 10-day quarantine and acclimatization period prior to the experimental procedures.

Animals were excluded if, before baseline measurements, they exhibited signs of clinical illness, congenital abnormalities that could affect cardiovascular or renal function, baseline health conditions that did not meet the eligibility criteria, anesthesia-related complications occurring before baseline data collection, or death before completion of all primary outcome measurements. Data points were excluded if blood samples could not be analyzed, measurement results were technically invalid, or data collection at time points T1, T2, or T3 was incomplete. In this study, all animals that completed the full experimental protocol were included in the final analysis, and no animals or data points were excluded from the final dataset. Laboratory personnel performing biomarker analyses were blinded to treatment allocation. Data analysis was conducted using coded datasets until completion of the primary statistical analyses.

This study was designed, conducted, and reported in accordance with the ARRIVE 2.0 guidelines for animal research. Continuous variables were displayed as mean ± standard deviation or median with interquartile range, where applicable. Group comparisons were executed using analysis of variance or nonparametric alternatives. Temporal fluctuations in biomarkers were evaluated by repeated-measures models. A two-tailed P value below 0.05 was considered statistically significant.

### 2.3 Data Analysis

Continuous variables were expressed as mean ± standard deviation (SD) for normally distributed data or median with interquartile range (IQR) for non-normally distributed data. Data normality was assessed using the Shapiro–Wilk test.

Comparisons of repeated measurements across time points (T1, T2, and T3) within each group were analyzed using repeated-measures ANOVA for normally distributed variables or the Friedman test for nonparametric data. Between-group comparisons were performed using one-way ANOVA or the Kruskal–Wallis test, as appropriate according to data distribution. Post hoc analyses with Bonferroni correction were applied when significant differences were identified.

All statistical tests were two-tailed, and a P value <0.05 was considered statistically significant. Statistical analyses were performed using IBM SPSS Statistics for Windows, Version 26.0 (IBM Corp., Armonk, NY, USA).

## 3. Results

### 3.1 Cystatin C

Repeated measurements of cystatin C demonstrated distinct temporal patterns among the three resuscitation strategy groups ( Table 1). In the fluid resuscitation group (Group 1), median cystatin C increased from baseline (T1: 1275.45 ng/mL) to septic shock (T2: 1354.5 ng/mL) and subsequently decreased post-intervention (T3: 843.6 ng/mL). A similar pattern was observed in the fluid plus norepinephrine group (Group 2), with a rise during septic shock (T2: 1768 ng/mL) compared with baseline (T1: 1702 ng/mL), followed by a decrease after intervention (T3: 1267.65 ng/mL).

**Table 1.** Cystatic-C level among groups.

Variable	T1	T2	T3	P value
Cystatin-C (ng/ml)				
Group 1 (Fluid)	1275,45 (910,9–1640)	1354,5 (1192–1517)	843,6 (715,2–972)	0,223 ^a^
Group 2 (NE + Fluid)	1702 (1131–2273)	1768 (1615–1921)	1267,65 (844,3–1691)	0,607 ^a^
Group 3 (NE)	1793 (522,9–3064)	1669,4 (503,8–2835)	1937,5 (1009–2866)	0,607 ^a^

Data are presented as median (min–max).

NE: Norepinephrine; T1: Before sepsis induction; T2: During septic shock; T3: After intervention

^a^ Friedman

In contrast, the early norepinephrine group (Group 3) showed a slight decrease during septic shock (T2: 1669.4 ng/mL) followed by an increase post-intervention (T3: 1937.5 ng/mL). Within-group comparisons using the Friedman test revealed no statistically significant temporal differences (P > 0.05).

### 3.2 NGAL

Plasma NGAL levels showed temporal fluctuations across groups ( Table 2). In Group 1, NGAL increased from baseline (T1: 37.47 mg/mL) to septic shock (T2: 43.81 mg/mL), then decreased after intervention (T3: 34.76 mg/mL). Group 2 demonstrated a similar trend. Conversely, Group 3 exhibited a decrease during septic shock followed by a post-intervention increase.

**Table 2.** NGAL level among groups.

Variable	T1	T2	T3	P value
*Neutrophil Gelatinase Associated Lipocalin* (NGAL) (ng/ml)				
Group 1 (Fluid)	37,47 (37,42–37,52)	43,81 (38,87–48,76)	34,76 (28,25–41,28)	0,867 ^a^
Group 2 (NE + Fluid)	39,47 (37,08–41,86)	45,37 (34,80–55,95)	39,35 (30,76–47,94)	0,223 ^a^
Group 3 (NE)	37,09 (17,92–56,27)	35,63 (16,56–54,71)	45,25 (34,83–55,68)	0,223 ^a^

Data are presented as median (min–max).

NE: Norepinephrine; T1: Before sepsis induction; T2: During septic shock; T3: After intervention

^a^ Friedman

### 3.3 Urine dynamics

Sequential ultrasonographic assessments of bladder volume showed variable trends among groups ( Table 3). Group 1 demonstrated an increase from T1 to T2 followed by a decrease at T3. Groups 2 and 3 showed progressive increases over time.

**Table 3.** Bladder volume among groups.

Variable	T1	T2	T3	P value
Urine Output (ml)				
Group 1 (Fluid)	242,500 (67,78–417,22)	833,520 (206,53–1460,51)	451,335 (269,25–633,42)	0,223 ^a^
Group 2 (NE + Fluid)	114,725 (61,17–168,28)	178,885 (126,96–230,81)	271,875 (262,89–280,89)	0,223 ^a^
Group 3 (NE)	108,540 (62,36–154,72)	228,510 (97,53–359,49)	316,795 (178,36–455,23)	0,135 ^a^

Data are presented as median (min–max).

NE: Norepinephrine; T1: Before sepsis induction; T2: During septic shock; T3: After intervention

^a^ Friedman

### 3.4 Serum creatinine

Serum creatinine levels remained stable across all time points in each group ( Table 4). Within-group analysis showed no significant temporal variation (P > 0.05). Intergroup comparisons were likewise not statistically significant.

**Table 4.** Creatinin serum levels among groups The differences in creatinine serums level between the septic shock phase and the post-intervention phase for each group.

Variable	T1	T2	T3	P value
Creatinine (mg/dL)				
Group 1 (Fluid)	1,50 (1,40–1,60)	1,50 (1,40–1,60)	1,50 (1,40–1,60)	0,000 ^a^
Group 2 (NE + Fluid)	1,45 (1,00–1,90)	1,45 (1,00–1,90)	1,45 (1,00–1,90)	0,000 ^a^
Group 3 (NE)	1,45 (1,30–1,60)	1,45 (1,30–1,60)	1,45 (1,30–1,60)	0,156 ^a^

Data are presented as median (min–max).

NE: Norepinephrine; T1: Before sepsis induction; T2: During septic shock; T3: After intervention

^a^ Friedman

## 4. Discussion

This experimental porcine study evaluated early renal biomarker dynamics during septic shock under three distinct resuscitation strategies. The principal finding was that structural biomarkers (NGAL and cystatin C) demonstrated earlier temporal changes compared with serum creatinine, although these differences did not reach statistical significance.

Cystatin C has been described as a sensitive marker of early glomerular filtration changes during acute inflammatory states such as sepsis. ^11^ ^–^ ^13^ Unlike creatinine, cystatin C production is relatively constant and less influenced by muscle mass or haemodilution, factors that are particularly relevant in critically ill patients undergoing aggressive fluid resuscitation (12). In the present study, fluid-based strategies were associated with post-intervention declines in cystatin C, whereas the early norepinephrine-dominant strategy demonstrated a delayed increase. These findings may reflect complex intrarenal hemodynamic responses to vasopressor therapy.

Norepinephrine is recommended as the first-line vasopressor in septic shock to restore mean arterial pressure ^7^ However, its renal effects remain debated. Experimental and clinical evidence suggests that while norepinephrine increases renal perfusion pressure, it may alter intrarenal microcirculatory distribution. ^14^ ^,^ ^15^ These mechanisms could partially explain the delayed cystatin C increase observed in the norepinephrine-only group.

NGAL exhibited similar directional trends, supporting previous reports that NGAL rises early in acute kidney injury and may precede creatinine elevation. ^16^ ^–^ ^18^ In contrast, serum creatinine remained unchanged across time points in all groups. This observation aligns with current literature demonstrating that creatinine is a delayed and relatively insensitive marker during the early phase of sepsis-associated acute kidney injury. ^4^ ^,^ ^12^ ^,^ ^19^ Creatinine kinetics are affected by haemodilution, altered production, and delayed equilibration following acute reductions in glomerular filtration rate. ^12^

Urine output, assessed indirectly using ultrasonographic bladder volume monitoring, showed variable trends without statistical significance. Ultrasonography has been proposed as a non-invasive tool for urine output assessment in critical care settings ^9^ ^,^ ^10^ although it does not allow precise quantification in mL/kg/h and cannot directly correspond to established acute kidney injury diagnostic thresholds. ^4^ ^,^ ^16^ ^,^ ^17^

The absence of statistically significant differences among resuscitation strategies should be interpreted cautiously. A limitation of this study is the relatively small sample size in each treatment group, which may have reduced the statistical power to detect significant intergroup differences and limited the assessment of variability within groups. Baseline differences observed among animals may reflect biological variability inherent to large-animal sepsis models. However, sample size determination was performed with particular emphasis on the reduction principle to minimize the number of experimental animals used. Increasing the sample size beyond the calculated requirement would raise important ethical concerns regarding animal welfare. Despite this limitation, the present study was intended as an exploratory translational model to evaluate biomarker dynamics and hemodynamic responses during septic shock resuscitation. Further studies with larger cohorts are warranted to validate these findings and improve statistical. Sepsis-associated acute kidney injury is multifactorial, involving inflammatory signaling pathways, endothelial dysfunction, and microcirculatory alterations beyond systemic hemodynamic variables alone. ^11^ ^,^ ^20^

Overall, these findings suggest that structural biomarkers may reflect early renal stress before conventional functional markers. Although early norepinephrine administration restored systemic hemodynamics, its renal biomarker profile did not conclusively demonstrate superiority. Larger studies with extended follow-up and combined functional–structural assessment are needed to clarify renal-protective resuscitation strategies.

## 6. Conclusions

In this porcine septic shock model, NGAL and cystatin C exhibited earlier dynamic changes than serum creatinine. Although differences among resuscitation strategies were not statistically significant, distinct biomarker patterns were observed. Larger studies with extended observation periods are required to clarify the renal impact of early fluid and vasopressor strategies.

## Ethics consideration

All procedures were approved by the Animal Ethics Committee School of Veterinary Medicine and Biomedical Science Institut Pertanian Bogor University number 266/KEH/SKE/X/2024.

## Data Availability

The datasets generated and analyzed during the current study are publicly available in the Zenodo repository at DOI: https://doi.org/10.5281/zenodo.20339160. ^21^ The repository contains the raw and processed data underlying the findings of this study, including plasma NGAL measurements, plasma cystatin C measurements, serum creatinine values, urinary bladder volume assessments, hemodynamic parameters, and the datasets used for statistical analyses. Data are available under the terms of the Creative Commons Attribution 4.0 International license (CC-BY 4.0). Zenodo: ARRIVE checklist for Early identification of sepsis-induced acute kidney injury utilizing NGAL, Cystatin C, urine output, and serum creatinine in a porcine (Sus scrofa) model of septic shock: A comparative study of fluid and vasopressor strategies. https://doi.org/10.5281/zenodo.20750365. ^21^ Data are available under the terms of the Creative Commons Attribution 4.0 International license (CC-BY 4.0).
