# Early Identification of Sepsis-induced Acute Kidney Injury Utilizing NGAL, Cystatin C, Urine Output, and Serum Creatinine in a Porcine ( *Sus scrofa*) Model of Septic Shock: A comparative Study of Fluid and Vasopressor Strategies

**DOI:** 10.12688/f1000research.182793.2

**Published:** 2026-11-05

**Authors:** Andriamuri Primaputra Lubis, Syah Mirsya Warli, Tasrif Hamdi, Gunanti Gunanti, Antonius Hocky Pudjiadi, Muhammad Rusda, Cut Meliza Zainumi, Raka Jati Prasetya

**Affiliations:** 1 Philosophy Doctor in Medicine Program, Faculty of Medicine, Universitas Sumatera Utara, Medan, North Sumatra, Indonesia; 2 Department of Anesthesiology and Intensive Care, Faculty of Medicine, Universitas Sumatera Utara Fakultas Kedokteran, Medan, North Sumatra, Indonesia; 3 Division of Urology, Department of Surgery, Universitas Sumatera Utara Fakultas Kedokteran, Medan, North Sumatra, Indonesia; 4 Division of Surgery and Radiology, School of Veterinary Medicine and Biomedical Sciences, Institut Pertanian Bogor, Bogor, West Java, Indonesia; 5 Department of Child Health, Faculty of Medicine, Universitas Indonesia, Cipto Mangunkusumo Hospital, Jakarta, Indonesia, Universitas Indonesia, Depok, West Java, Indonesia; 6 Department of Obstetrics and Gynecology, Universitas Sumatera Utara Fakultas Kedokteran, Medan, North Sumatra, Indonesia

**Keywords:** Sepsis; Acute Kidney Injury; Neutrophil Gelatinase-Associated Lipocalin; Cystatin C; Norepinephrine

## Abstract

**Background:**

Sepsis-associated acute kidney injury (SA-AKI) contributes significantly to ICU morbidity and mortality, yet early detection remains challenging due to the delayed response of conventional markers such as serum creatinine and urine output. This study aimed to evaluate the temporal patterns of early renal biomarkers—neutrophil gelatinase-associated lipocalin (NGAL) and cystatin C—compared with conventional functional markers in a porcine model of septic shock under three fluid and vasopressor resuscitation strategies.

**Methods:**

In a randomized experimental study, six Sus scrofa pigs underwent standardized septic shock induction and were allocated into three resuscitation groups: Ringer’s lactate (30 mL/kg), fluid combined with early norepinephrine infusion, or early norepinephrine with maintenance fluids. Plasma NGAL, cystatin C, serum creatinine, and ultrasonographically assessed urine dynamics were measured sequentially alongside continuous hemodynamic monitoring. Data were analyzed using repeated-measures and nonparametric tests (p < 0.05).

**Results:**

NGAL and cystatin C exhibited numerical temporal fluctuations across observation periods and differing trajectories among resuscitation strategies. Post-intervention decreases in NGAL and cystatin C coccurred in fluid-based groups, whereas early norepinephrine administration was followed by numerical increases. However, neither between-group nor within-group temporal changes reached statistical significance for NGAL, cystatin C, or serum creatinine.

**Conclusion:**

In this exploratory pilot study, NGAL and cystatin C displayed numerical temporal shifts during septic shock; however, these changes lacked statistical significance and cannot establish early SA-AKI detection. Given the unstandardized urine output criteria and non-significant creatinine kinetics, these findings represent exploratory evidence of renal biomarker dynamics rather than definitive diagnostic proof of SA-AKI.

## 1. Introduction

Sepsis is a critical organ dysfunction resulting from an aberrant host response to infection and continues to be a predominant cause of morbidity and mortality globally. ^1^ Sepsis-associated acute kidney injury (SA-AKI) is one of the most prevalent and severe complications of septic shock, independently linked to extended intensive care unit (ICU) stay, progression to chronic kidney disease, and heightened mortality rates. ^2, 3^ Despite advancements in sepsis management, the prompt identification of renal injury during the initial resuscitation phase poses a significant clinical challenge.

The current diagnostic criteria for acute kidney injury predominantly rely on functional markers, notably serum creatinine levels and urine output. ^4^ However, serum creatinine serves as a delayed and insensitive indicator of acute changes in glomerular filtration, particularly in critically ill patients undergoing fluid loading, haemodilution, altered muscle metabolism, and systemic inflammation. Neurohormonal responses, hemodynamic therapies, and fluid balance may all impact urine output, making it less reliable as a standalone sign of kidney damage. As a result, considerable structural damage to the kidneys may occur before traditional criteria for Acute Kidney Injury (AKI) are met.

Biomarkers reflecting renal tubular injury and glomerular filtration have emerged as potential adjuncts to conventional functional measures of kidney function. NGAL is associated with tubular injury and inflammatory responses, whereas cystatin C primarily reflects glomerular filtration and may be less affected by muscle mass than creatinine. ^4^ Neutrophil gelatinase–associated lipocalin (NGAL) is swiftly upregulated and released by damaged renal tubular epithelial cells following ischemic or inflammatory insults. ^4^ Cystatin C, a low–molecular weight protease inhibitor that is freely filtered by the glomerulus, offers a more stable indication of glomerular filtration independent of muscle mass and volume status. ^2, 3^ Collectively, these biomarkers may facilitate earlier detention of renal injury prior to the manifestation of significant functional decline.

Fluid resuscitation and vasopressor treatment are the primary strategies for hemodynamic care in septic shock. ^3, 4^ Current recommendations recommend the immediate administration of intravenous crystalloids, followed by vasopressors, to maintain adequate mean arterial pressure. However, aggressive fluid resuscitation may exacerbate endothelial dysfunction, interstitial edema, and renal congestion, ultimately worsening kidney damage. Conversely, the immediate infusion of norepinephrine may restore vascular tone, improve organ perfusion, and reduce excessive fluid exposure. The renal consequences of diverse fluid–vasopressor strategies during the first phase of septic shock, particularly with early kidney damage indicators, remain little defined.

Experimental animal models provide a controlled environment to investigate the pathophysiological mechanisms of SA-AKI and evaluate renal biomarkers under standardized hemodynamic conditions. The porcine (Sus scrofa) model is especially suitable due to its significant anatomical, physiological, and hemodynamic resemblances to humans. We hypothesized that NGAL and cystatin C would demonstrate earlier temporal changes than conventional renal parameters during septic shock and resuscitation. Therefore, the aim of this exploratory study was to characterize temporal changes in NGAL and cystatin C in comparison with serum creatinine and ultrasonographically assessed bladder volume under three different fluid and vasopressor resuscitation strategies. Therefore, the aim of this study was to compare early biomarkers (NGAL and cystatin C) with conventional functional markers (serum creatinine and urine output) in a porcine model of septic shock, and to evaluate their temporal patterns under three different fluid and vasopressor resuscitation strategies.

## 2. Methods

### 2.1 Study Design

This study was a randomized, controlled experiment using a porcine model of septic shock ^5^ included in the study and randomly allocated to three treatment groups (n = 2 animals per group). Given the limited number of animals, this study was designed as an exploratory pilot study to characterize early renal biomarker trajectories following different resuscitation strategies in experimental septic shock. All procedures were approved by the Institutional Animal Care and Use Committee and and adhered to national regulations for laboratory animal research, including the 3Rs (Reduction, Replacement, Refinement) and 5Fs (Freedom from Hunger and Thirst, Freedom from Discomfort, Freedom from Pain, Injury or Disease, Freedom to Express Normal Behavior, Freedom from Fear and Distress). ^6^ The experimental unit was an individual animal (one pig). Six animals were.

### 2.2 Data Collection

Measures were implemented to mitigate animal suffering and decrease the number of animals utilized. In this study, the experimental animal model used consisted of male pigs ( *Sus scrofa*) aged fourteen to eighteen weeks, weighing between 55 and 60 kg. Before the trial, six healthy The sample size used in this study was based on the resource equation method. The study consisted of one control group and three treatment groups, with three repeated measurements performed at different time points: before sepsis induction, during sepsis/septic shock, and after therapy.

Sample size was determined prior to the experiment by considering the Reduction principle in large-animal research and applying the resource equation method. As this was an exploratory preclinical/translational study, the number of animals was kept to the minimum necessary while maintaining the ability to evaluate temporal changes in renal biomarkers and hemodynamic parameters in a septic shock model.

Six male pigs (Sus scrofa) were used as experimental units and were equally allocated to three resuscitation strategy groups (n = 2 per group): (1) a 30 mL/kg lactated Ringer’s group, (2) a 30 mL/kg lactated Ringer’s plus early norepinephrine group, and (3) an early norepinephrine group receiving maintenance lactated Ringer’s at 3 mL/kg/h. The analyzed sample size (n) for each outcome is reported in the Results tables. No animals, experimental units, or data points were excluded from the final analysis, provided that all measurements at T1, T2, and T3 were available.

Sus scrofa pigs of similar age and body weight were acclimatized. The animals were not allowed to eat for a night, but they may drink as much water as they desired. Each experimental animal was anesthetized with atropine sulfate 0.04 mg/kg body weight intramuscularly, ketamine HCl 10% at a dose of 20 mg/kg body weight intramuscularly, and xylazine HCl 2% at a dose of 2 mg/kg body weight intramuscularly. Anesthesia was maintained with propofol at a dose of 2–4 mg/kg body weight intravenously, supported by mechanical ventilation using a volume-controlled mode adjusted according to arterial blood gas analysis. Normal saline 0.9% at a dose of 3 mL/kg/hour was administered for maintenance fluid therapy.

After endotracheal intubation, the participants were mechanically ventilated with oxygen-air mixtures to keep the levels of oxygen and carbon dioxide in the blood normal. During the whole trial, heart rate, blood pressure, oxygen saturation, and temperature were all watched all the time. One hour after stabilization, hemodynamic parameters, which also served as indicators of volume adequacy, and blood samples were obtained for baseline measurements. This procedure was performed through the right femoral artery using an aseptic technique, and a thermistor-tipped transpulmonary thermodilution catheter (PiCCO, PULSION Medical Systems SE, Munich, Germany) was inserted into the right femoral artery for hemodynamic monitoring and temperature measurement.

Data collection and measurements were performed at three predefined time points: T1, during baseline data acquisition after the stabilization period; T2, at the time of sepsis induction/septic shock; and T3, after the post-resuscitation intervention. Hemodynamic parameters, laboratory examinations, and renal function assessments were evaluated at each time point to determine the physiological changes occurring throughout the experimental protocol.

Sepsis was induced in the experimental animals by administration of *Escherichia coli* endotoxin (lipopolysaccharide 0111: B4, Difco Laboratories, Detroit, MI) via continuous infusion initiated at a rate of 0.30 μg/kg/hour. Septic shock was considered established when evidence of organ dysfunction was present, indicated by a mean arterial pressure (MAP) ≤ 65 mmHg sustained for 30 minutes. ^6^

There were three treatment groups, and animals were randomly put into one of them. The Fluid Resuscitation Group: Animals received Ringer’s lactate at a dose of 30 mL/kg administered over 30 to 60 minutes, in accordance with contemporary sepsis resuscitation guidelines. ^7^ The Fluid plus Early Norepinephrine Group: Animals received Ringer’s lactate at a dose of 30 mL/kg along with a quick start to norepinephrine infusion, which started at 0.1 μg/kg/min and was changed to keep the mean arterial pressure at least 65 mmHg. If the MAP had not reached ≥65 mmHg, a 500 mL fluid bolus was administered in addition to norepinephrine (NE) at 0.2 μg/kg/minute. If the MAP remained <65 mmHg, the norepinephrine dose was further titrated up to 0.3 μg/kg/minute at each hemodynamic assessment until the target MAP ≥65 mmHg was achieved. Failure to achieve the target MAP despite these interventions was considered treatment failure. ^7^ The Early Norepinephrine with Maintenance Fluid Group: Animals received norepinephrine starting at 0.1 μg/kg/min from the beginning of septic shock, along with maintenance Ringer’s lactate infusion at 3 mL/kg/h. This was an early vasopressor approach meant to reduce fluid overload. ^7, 8^ Hemodynamic targets were maintained throughout the observation period with regular adjustments of fluid administration and vasopressor dose.

Following a 10-day quarantine and acclimatization period, animals that were deemed healthy and met all study eligibility criteria were assigned sequential individual identification codes (P01–P06). Allocation to the study groups was performed using computer-based randomization with the RANDOM.ORG List Randomizer. The randomization list consisted of six allocation labels in a 1:1:1 ratio: two labels for the lactated Ringer’s group, two labels for the lactated Ringer’s plus early norepinephrine group, and two labels for the early norepinephrine with maintenance fluid group.

The allocation sequence was generated before the experiment by an independent staff member who was not involved in administering the interventions, measuring outcomes, performing biomarker assessments, conducting ultrasonographic examinations, or carrying out statistical analyses. To maintain allocation concealment, the randomization list was stored separately in a secure digital file accessible only to the staff member responsible for group allocation. Group assignments were revealed only after the animals had completed the acclimatization period, met all eligibility criteria, and undergone baseline measurements.

Potential confounding factors were minimized by using animals of the same sex with similar ages and body weight ranges; housing all animals in the same facility under individual housing conditions; applying identical quarantine and acclimatization procedures; and standardizing anesthesia, mechanical ventilation, septic shock induction, and hemodynamic monitoring protocols. Outcome measurements were performed at the predefined time points (T1, T2, and T3) in accordance with the study protocol.

Blood samples were collected at predetermined intervals to assess plasma NGAL, cystatin C, and serum creatinine, which are frequently employed biomarkers for the evaluation of sepsis-associated acute kidney injury. NGAL and cystatin C biomarker concentrations were measured using the enzyme-linked immunosorbent assay (ELISA) method with a particle-enhanced turbidimetric immunoassay technique, in accordance with the manufacturers’ instructions. All samples were handled and assessed in a blind manner.

Bladder volume was assessed by ultrasonography at T1, T2, and T3 as an indirect measure of urinary accumulation. The urinary bladder was visualized using a standardized ultrasound approach, and bladder dimensions were used to estimate the intravesical volume. The bladder was not emptied between the serial ultrasound assessments; therefore, the measurements represented the accumulated bladder volume at each time point rather than urine production over a defined time interval. At the end of the experimental protocol, following euthanasia, the bladder was opened and the accumulated urine was completely collected and directly measured in milliliters. Because urine was not continuously collected and the measured volume was not normalized to body weight and time, the values cannot be interpreted as urine output in mL/kg/h and were not used to diagnose AKI according to KDIGO urine-output criteria. ^9, 10^

Blood samples were collected at predetermined intervals to assess plasma neutrophil gelatinase-associated lipocalin (NGAL), cystatin C, and serum creatinine as biomarkers of sepsis-associated acute kidney injury. Plasma NGAL concentrations were measured using a Porcine NGAL ELISA Kit (Fine Test, Wuhan Fine Biotech Co., Ltd., Wuhan, China; Catalog No. EP0121), while plasma cystatin C concentrations were measured using a Porcine Cystatin C (Cys-C) ELISA Kit (Fine Test, Wuhan Fine Biotech Co., Ltd., Wuhan, China; Catalog No. EP0046). Both biomarkers were quantified using a sandwich enzyme-linked immunosorbent assay (ELISA) technique according to the manufacturer’s instructions. All samples were processed under identical laboratory conditions and analyzed in a blinded manner to minimize measurement bias. Biomarker analyses were performed using the same lot of assay kits to reduce inter-assay variability.

Sepsis was induced by continuous intravenous infusion of Escherichia coli endotoxin (lipopolysaccharide O111:B4; Difco Laboratories, Detroit, MI, USA). Animals received Ringer’s lactate solution for fluid resuscitation and norepinephrine infusion for hemodynamic support according to group allocation. Anesthesia was induced using atropine sulfate (0.04 mg/kg intramuscularly), ketamine hydrochloride 10% (20 mg/kg intramuscularly), and xylazine hydrochloride 2% (2 mg/kg intramuscularly), and maintained with intravenous propofol (2–4 mg/kg). Mechanical ventilation was provided throughout the experimental period. At the completion of the experiment, all animals were humanely euthanized under deep general anesthesia. Anesthesia was maintained with continuous intravenous infusion of propofol (6–10 mg/kg/h) and fentanyl (5–25 μg/kg/h). Adequate anesthetic depth was confirmed before euthanasia by the absence of spontaneous movement, loss of jaw tone and corneal reflexes, and stable respiratory and hemodynamic parameters.

Euthanasia was performed by slow intravenous administration of magnesium sulfate (MgSO _4_) 50% injection (500 mg/mL; Magnesium Sulfate Heptahydrate 50% w/v Injection, Pfizer/Hospira, Lake Forest, IL, USA) at a dose of 200–250 mg/kg body weight until cardiac arrest occurred. Mechanical ventilation and physiologic monitoring were maintained throughout the procedure. Death was confirmed by the absence of pulse, heart sounds, respiratory sounds, spontaneous respiration, corneal reflexes, and response to painful stimuli. Monitoring was continued until cardiac and respiratory arrest were sustained. If any signs of life persisted, additional euthanasia measures were performed under veterinary supervision until death was confirmed.

All euthanasia procedures were performed by or under the supervision of a licensed veterinarian and were conducted in accordance with the AVMA Guidelines for the Euthanasia of Animals: 2020 Edition. Consistent with these guidelines, magnesium sulfate was administered only to animals that were unconscious and maintained under deep general anesthesia. Inclusion and exclusion criteria were established before the study commenced. Eligible animals were clinically healthy male pigs (Sus scrofa), aged 14–18 weeks, weighing 55–60 kg, with relatively homogeneous age and body weight ranges, and having completed a 10-day quarantine and acclimatization period prior to the experimental procedures.

Animals were excluded if, before baseline measurements, they exhibited signs of clinical illness, congenital abnormalities that could affect cardiovascular or renal function, baseline health conditions that did not meet the eligibility criteria, anesthesia-related complications occurring before baseline data collection, or death before completion of all primary outcome measurements. Data points were excluded if blood samples could not be analyzed, measurement results were technically invalid, or data collection at time points T1, T2, or T3 was incomplete. In this study, all animals that completed the full experimental protocol were included in the final analysis, and no animals or data points were excluded from the final dataset. Laboratory personnel performing biomarker analyses were blinded to treatment allocation. Data analysis was conducted using coded datasets until completion of the primary statistical analyses. Given the exploratory pilot design and the small number of experimental units (n = 2 per group), statistical analyses were considered descriptive and hypothesis-generating rather than confirmatory. Individual animal trajectories were considered alongside group-level summaries, and no inference regarding superiority of any resuscitation strategy was made.

This study was designed, conducted, and reported in accordance with the ARRIVE 2.0 guidelines for animal research. Continuous variables were displayed as mean ± standard deviation or median with interquartile range, where applicable. Group comparisons were executed using analysis of variance or nonparametric alternatives. Temporal changes within each treatment group were assessed descriptively and, where appropriate, using the Friedman test for repeated measurements. Given the very small number of experimental units per group (n = 2), inferential statistical results were considered exploratory and hypothesis-generating rather than confirmatory.

### 2.3 Data Analysis

Continuous variables were expressed as mean ± standard deviation (SD) for normally distributed data or median with interquartile range (IQR) for non-normally distributed data. Data normality was assessed using the Shapiro–Wilk test.

Comparisons of repeated measurements across time points (T1, T2, and T3) within each group were analyzed using repeated-measures ANOVA for normally distributed variables or the Friedman test for nonparametric data. Between-group comparisons were performed using one-way ANOVA or the Kruskal–Wallis test, as appropriate according to data distribution. Post hoc analyses with Bonferroni correction were applied when significant differences were identified.

All statistical tests were two-tailed, and a P value <0.05 was considered statistically significant. Statistical analyses were performed using IBM SPSS Statistics for Windows, Version 26.0 (IBM Corp., Armonk, NY, USA).

## 3. Results

### 3.1 Cystatin C

Repeated measurements of cystatin C demonstrated distinct temporal patterns among the three resuscitation strategy groups ( Table 1). In the fluid resuscitation group (Group 1), median cystatin C increased from baseline (T1: 1275.45 ng/mL) to septic shock (T2: 1354.5 ng/mL) and subsequently decreased post-intervention (T3: 843.6 ng/mL). A similar pattern was observed in the fluid plus norepinephrine group (Group 2), with a rise during septic shock (T2: 1768 ng/mL) compared with baseline (T1: 1702 ng/mL), followed by a decrease after intervention (T3: 1267.65 ng/mL).

**Table 1.** Temporal changes in cystatin C within each resuscitation strategy group.

Variable	T1	T2	T3	P *value*
Cystatin-C (ng/ml)				
Group 1 (Fluid)	1275,45 (910,9–1640)	1354,5 (1192–1517)	843,6 (715,2–972)	0,223 ^a^
Group 2 (NE + Fluid)	1702 (1131–2273)	1768 (1615–1921)	1267,65 (844,3–1691)	0,607 ^a^
Group 3 (NE)	1793 (522,9–3064)	1669,4 (503,8–2835)	1937,5 (1009–2866)	0,607 ^a^

P values represent within-group comparisons across T1, T2, and T3 using the Friedman test. Values are presented as median (minimum–maximum). No P value represents a between-group comparison.

^a^ Friedman.

Data are presented as median (min–max).

NE: Norepinephrine; T1: Before sepsis induction; T2: During septic shock; T3: After intervention.

In contrast, the early norepinephrine group (Group 3) showed a slight decrease during septic shock (T2: 1669.4 ng/mL) followed by an increase post-intervention (T3: 1937.5 ng/mL). Within-group comparisons using the Friedman test revealed no statistically significant temporal differences (P > 0.05).

### 3.2 NGAL

Plasma NGAL levels showed temporal fluctuations across groups ( Table 2). In Group 1, NGAL increased from baseline (T1: 37.47 Ng/mL) to septic shock (T2: 43.81 ng/mL), then decreased after intervention (T3: 34.76 ng/mL). Group 2 demonstrated a similar trend. Conversely, Group 3 exhibited a decrease during septic shock followed by a post-intervention increase.

**Table 2.** NGAL level among groups.

Variable	T1	T2	T3	P *value*
*Neutrophil Gelatinase Associated Lipocalin* (NGAL) (ng/ml)				
Group 1 (Fluid)	37,47 (37,42 – 37,52)	43,81 (38,87 – 48,76)	34,76 (28,25 – 41,28)	0,867 ^a^
Group 2 (NE + Fluid)	39,47 (37,08 – 41,86)	45,37 (34,80 – 55,95)	39,35 (30,76 – 47,94)	0,223 ^a^
Group 3 (NE)	37,09 (17,92 – 56,27)	35,63 (16,56 – 54,71)	45,25 (34,83 – 55,68)	0,223 ^a^

^a^ Friedman.

Data are presented as median (min–max).

NE: Norepinephrine; T1: Before sepsis induction; T2: During septic shock; T3: After intervention.

### 3.3 Urine Dynamics

Sequential ultrasonographic assessments demonstrated variable bladder-volume trajectories among the three groups. Because these measurements represented accumulated bladder volume rather than standardized urine production, they were not interpreted according to KDIGO urine-output criteria ( Table 3).

**Table 3.** Ultrasonographically estimated bladder volume (mL).

Variable	T1	T2	T3	P *value*
Urine Output (ml)				
Group 1 (Fluid)	242,500 (67,78 – 417,22)	833,520 (206,53 – 1460,51)	451,335 (269,25 – 633,42)	0,223 ^a^
Group 2 (NE + Fluid)	114,725 (61,17 – 168,28)	178,885 (126,96 – 230,81)	271,875 (262,89 – 280,89)	0,223 ^a^
Group 3 (NE)	108,540 (62,36 – 154,72)	228,510 (97,53 – 359,49)	316,795 (178,36 – 455,23)	0,135 ^a^

^a^ Friedman.

Data are presented as median (min–max).

NE: Norepinephrine; T1: Before sepsis induction; T2: During septic shock; T3: After intervention.

### 3.4 Serum Creatinine

Serum creatinine levels vary across all time points in each group ( Table 4). Within-group analysis showed no significant temporal variation (P > 0.05). Intergroup comparisons were likewise not statistically significant.

**Table 4.** Creatinin serum levels among groups. The differences in creatinine serums level between the septic shock phase and the post-intervention phase for each group.

Variable	T1	T2	T3	P *value*
Creatinine (mg/dL)				
Group 1 (Fluid)	1,30 (1,20-1,40)	1,80 (1,80-1,80)	1,10 (1,00-1,20)	0,156 ^a^
Group 2 (NE + Fluid)	1,20 (1,20-1,20)	1,65 (1,40-1,90)	1,20 (1,20-1,20)	0,135 ^a^
Group 3 (NE)	1,45 (1,30-1,60)	1,70 (1,40-2,00)	2,05 (1,40-2,70)	0,156 ^a^

^a^ Friedman.

Data are presented as median (min–max).

NE: Norepinephrine; T1: Before sepsis induction; T2: During septic shock; T3: After intervention.

## 4. Discussion

This experimental porcine study evaluated early renal biomarker dynamics during septic shock under three distinct resuscitation strategies. Given the exploratory nature and very small sample size (n = 2 per group), analyses were primarily descriptive, with emphasis on individual animal trajectories and changes over time. Between-group statistical comparisons were interpreted cautiously and were considered hypothesis-generating rather than confirmatory. The principal observation of this exploratory study was that NGAL and cystatin C exhibited numerical temporal fluctuations during septic shock and after resuscitation, while serum creatinine showed comparatively different temporal patterns. However, none of the biomarker changes reached statistical significance, and the study was not powered to establish differences among treatment strategies. Therefore, these observations should be considered hypothesis-generating rather than evidence that NGAL or cystatin C can provide earlier diagnosis of acute kidney injury.

Cystatin C has been described as a sensitive marker of early glomerular filtration changes during acute inflammatory states such as sepsis. ^11– 13^ Unlike creatinine, cystatin C production is relatively constant and less influenced by muscle mass or haemodilution, factors that are particularly relevant in critically ill patients undergoing aggressive fluid resuscitation. ^12^ In the present study, fluid-based strategies were associated with post-intervention declines in cystatin C, whereas the early norepinephrine-dominant strategy demonstrated a delayed increase. These findings may reflect complex intrarenal hemodynamic responses to vasopressor therapy.

Norepinephrine is recommended as the first-line vasopressor in septic shock to restore mean arterial pressure. ^7^ However, its renal effects remain debated. Experimental and clinical evidence suggests that while norepinephrine increases renal perfusion pressure, it may alter intrarenal microcirculatory distribution. ^14, 15^ The delayed increase in cystatin C observed in the early norepinephrine plus maintenance fluid group may reflect the complex relationship between systemic hemodynamics and intrarenal perfusion. Restoration of systemic MAP with norepinephrine does not necessarily imply normalization of renal microcirculatory perfusion, as vasoconstrictor therapy may alter intrarenal vascular distribution. However, because only two animals were included in this group and the observed cystatin C change was not statistically significant, this interpretation remains hypothesis-generating rather than a demonstrated mechanism.

NGAL exhibited numerical temporal fluctuations across the three treatment groups. Although NGAL has been reported to increase before serum creatinine in several clinical and experimental settings, ^16– 18^ the present study did not demonstrate a statistically significant temporal increase in NGAL. Therefore, the observed changes should be interpreted as potential early renal biomarker responses rather than evidence of established AKI or diagnostic superiority over serum creatinine. ^16– 18^ Serum creatinine varies across time points in all groups. This observation aligns with current literature demonstrating that creatinine is a delayed and relatively insensitive marker during the early phase of sepsis-associated acute kidney injury. ^4, 12, 19^ Creatinine kinetics are affected by haemodilution, altered production, and delayed equilibration following acute reductions in glomerular filtration rate. ^12^

Urine output, assessed indirectly using ultrasonographic bladder volume monitoring, showed variable trends without statistical significance. Ultrasonography has been proposed as a non-invasive tool for urine output assessment in critical care settings ^9, 10^ although it does not allow precise quantification in mL/kg/h and cannot directly correspond to established acute kidney injury diagnostic thresholds. ^4, 16, 17^ Because serum creatinine levels vary and urine output was not assessed according to standardized KDIGO criteria, our findings should not be interpreted as definitive evidence of AKI. Rather, the observed changes in NGAL and cystatin C may represent early renal responses to septic shock that precede detectable changes in conventional functional markers. Accordingly, the present study should not be interpreted as demonstrating KDIGO-defined acute kidney injury. Rather, the experimental protocol demonstrated septic shock accompanied by temporal changes in circulating renal biomarkers. In the absence of standardized urine output measurements and a sustained creatinine-based KDIGO criterion, these findings are more appropriately interpreted as early renal biomarker responses or renal stress during septic shock.

This exploratory pilot study demonstrated numerical temporal changes in NGAL and cystatin C during septic shock under different resuscitation strategies. However, these changes were not statistically significant, and the study did not establish KDIGO-defined acute kidney injury because standardized urine-output criteria were not available and serum creatinine did not demonstrate statistically significant temporal changes. Therefore, the findings should be interpreted as hypothesis-generating observations of early renal biomarker dynamics rather than evidence that NGAL or cystatin C can provide earlier diagnosis of sepsis-associated acute kidney injury. Larger adequately powered studies incorporating standardized urine-output measurements and combined structural and functional renal endpoints are required to determine the diagnostic value of these biomarkers. Several limitations should be considered. First, the study included only six animals, with two animals per treatment group, substantially limiting statistical power and precluding reliable inference regarding treatment superiority. Second, the observation period was limited and may not have captured delayed changes in serum creatinine or later biomarker kinetics. Third, urine production was not measured by continuous urinary catheterization and therefore could not be expressed as mL/kg/h or used to establish KDIGO urine-output criteria. Instead, ultrasonographic bladder-volume measurements and terminal bladder urine collection were used, which represent urinary accumulation rather than standardized urine production. Fourth, renal histopathological examination was not performed, preventing direct correlation between biomarker changes and microscopic structural injury. Fifth, physiological and biological differences between pigs and humans may limit extrapolation of the findings to clinical sepsis. Sixth, anesthesia and terminal experimental procedures may influence renal hemodynamics and should be considered when interpreting renal physiology. Finally, the study was designed as an exploratory pilot experiment, and the observed biomarker trajectories should therefore be regarded as hypothesis-generating rather than generalizable evidence.

Despite this limitation, the present study was intended as an exploratory translational model to evaluate biomarker dynamics and hemodynamic responses during septic shock resuscitation. Further studies with larger cohorts are warranted to validate these findings and improve statistical. Sepsis-associated acute kidney injury is multifactorial, involving inflammatory signaling pathways, endothelial dysfunction, and microcirculatory alterations beyond systemic hemodynamic variables alone. ^11, 20^

Overall, these findings suggest that biomarkers may reflect early renal stress before conventional functional markers. Although early norepinephrine administration restored systemic hemodynamics, its renal biomarker profile did not conclusively demonstrate superiority. Larger studies with extended follow-up and combined functional–structural assessment are needed to clarify renal-protective resuscitation strategies. It is important to distinguish the biological characteristics of the biomarkers evaluated in this study. NGAL is primarily considered a marker associated with tubular injury and stress, whereas cystatin C is predominantly a marker of glomerular filtration. Accordingly, the two biomarkers should not be classified collectively as structural biomarkers. Their temporal changes may reflect different components of the renal response to septic shock.

## 5. Conclusions

This exploratory pilot study identified differences in the temporal patterns of renal biomarkers across resuscitation strategies, although NGAL and cystatin C showed numerical changes before serum creatinine. This exploratory pilot study demonstrated numerical temporal changes in NGAL and cystatin C during septic shock under different resuscitation strategies. However, these changes were not statistically significant, and the study did not establish KDIGO-defined acute kidney injury because standardized urine-output criteria were not available and serum creatinine did not demonstrate statistically significant temporal changes. Therefore, the findings should be interpreted as hypothesis-generating observations of early renal biomarker dynamics rather than evidence that NGAL or cystatin C can provide earlier diagnosis of sepsis-associated acute kidney injury. Larger adequately powered studies incorporating standardized urine-output measurements and combined structural and functional renal endpoints are required to determine the diagnostic value of these biomarkers. Larger studies with extended observation periods are required to clarify the renal impact of early fluid and vasopressor strategies.

## Declarations

### Ethics consideration

All procedures were approved by the Animal Ethics Committee School of Veterinary Medicine and Biomedical Science Institut Pertanian Bogor University number 266/KEH/SKE/X/2024.

## Author’s Contributions

Conceptualization: APL, SMW, TH, GG; Data Curation: SMW, TH, AP; Formal Analysis: APL, TH, GG, AP; Funding Acquisition: APL, AP, MR; Investigation: SMW, AP, CMZ; Methodology: APL, MR, RJP, GG; Project Administration: TH, RJP; Resources: GG, AP; Software: TH, CMM; Supervision: APL, RJP; Validation: APL, SMW; Visualization: CMZ; Writing – Original Draft Preparation: APL, MR; Writing - Review & Editing: APL, SMW, TH.

## Data Availability

The datasets generated and analyzed during the current study are publicly available in the Zenodo repository at DOI: https://doi.org/10.5281/zenodo.20339160. ^21^ The repository contains the raw and processed data underlying the findings of this study, including plasma NGAL measurements, plasma cystatin C measurements, serum creatinine values, urinary bladder volume assessments, hemodynamic parameters, and the datasets used for statistical analyses. The data are openly accessible and may be reused in accordance with the license assigned to the repository.
