# Supplementary material for: Early Identification of Sepsis-induced Acute Kidney Injury Utilizing NGAL, Cystatin C, Urine Output, and Serum Creatinine in a Porcine ( Sus scrofa) Model of Septic Shock: A comparative Study of Fluid and Vasopressor Strategies
Source: F1000Res. 2026 Nov 5;15:1077. Originally published 2026 Jul 6. [Version 2] doi: 10.12688/f1000research.182793.2 (PMC13770168; doi:10.12688/f1000research.182793.2)
Supplement: Peer review history

# Reviewer report: Reviewer response for version 2

**DOI:** 10.5256/f1000research.212175.r538577

**Published:** 2026-11-14

**Authors:** Rohini Saha

**Affiliations:** 1 All India Institute of Medical Sciences, New Delhi, Delhi, India

The ammends are correct. I do not have any further comment.

Is the work clearly and accurately presented and does it cite the current literature?

Yes

If applicable, is the statistical analysis and its interpretation appropriate?

No

Are all the source data underlying the results available to ensure full reproducibility?

No

Is the study design appropriate and is the work technically sound?

No

Are the conclusions drawn adequately supported by the results?

No

Are sufficient details of methods and analysis provided to allow replication by others?

No

Reviewer Expertise:

Liver biology, Renal Biology, Biomarkers, ACLF, Immunology, Neutrophil

I confirm that I have read this submission and believe that I have an appropriate level of expertise to confirm that it is of an acceptable scientific standard.

# Reviewer report: Reviewer response for version 1

**DOI:** 10.5256/f1000research.201765.r512626

**Published:** 2026-09-04

**Authors:** Robert Kalyesubula, Odong Christopher

**Affiliations:** 1 Makerere University College of Health Sciences, Kampala, Uganda; 2 Internal Medicine, Mulago Naonal Specialist Hospital, Kampala, Central, Uganda

We thank the author for conducting this study and for the opportunity to review it.

 The study, entitled “ *Early identification of sepsis-induced acute kidney injury utilizing NGAL, Cystatin C, urine output, and serum creatinine in a porcine (Sus scrofa) model of septic shock: A comparative study of fluid and vasopressor strategies*,” aims to compare early structural biomarkers (NGAL and cystatin C) with conventional functional markers (serum creatinine and urine output) in a porcine model of septic shock, and to examine their temporal patterns under three different fluid and/or vasopressor resuscitation strategies. The study of how NGAL, cystatin C, serum creatinine, and bladder volume change over time during septic shock in a pig model, under different fluid and norepinephrine resuscitation techniques, is clinically relevant, as there remains difficulty in the early detection of sepsis-associated acute kidney injury.

 We have identified several points that need to be addressed;

**1: TITLE: “** *Early identification of sepsis-induced acute kidney injury utilizing NGAL, Cystatin C, urine output, and serum creatinine in a porcine (Sus scrofa) model of septic shock: A comparative study of fluid and vasopressor strategies* *” *is clear on point and effectively engages the reader

**2: ABSTRACT**

**Background:** The novelty of the study is not sufficiently emphasized. Clearly articulate the investigation’s specific contribution to the existing knowledge gap.

**Methods:** The methods section is well written and concise.

**Results:** All results should be consistently supported by statistical findings from Tables and figure legends. Reporting results without the corresponding statistics is insufficient. Please add statistics to support the current statements.

**Conclusion: **The key findings should be stated clearly and, where appropriate, be placed in context by referring to previous studies.

 The abstract as a whole need to be revised since it does clearly summarize the study.

**MAIN BODY OF THE ARTICLE**

**INTRODUCTION**

 Well written, clear, and to the point. However, knowledge gaps and the novelty of the work should be more clearly emphasized, with reference to data from previous investigations.

**METHOD**

 1. Please clarify how you defined acute kidney injury. While the study focuses on sepsis-associated AKI, most outcomes reported are biomarker concentrations and changes in bladder volume. The manuscript notes that bladder ultrasonography did not provide urine output in the standard mL/kg/h format, so it could not be directly compared with established AKI diagnostic thresholds. Did you use any predefined criteria to diagnose AKI, or was the study only intended to examine changes in renal biomarkers?

 2. Since bladder volume was employed as an indirect indicator of urine dynamics, more details should be given in the methods section regarding how changes in bladder volume were understood to reflect urine production. You must include the ultrasonographic measurement procedure, when the measurements were taken, and your interpretation of the changes in bladder volume. It is also necessary to address the limitations of using this surrogate measure in the Results and Discussion sections. Lastly, since the manuscript mentioned three treatment groups, was there actually a control group? Please make this clear.

 3. The manuscript states that the sample size was determined using the resource equation method and the principle of reduction. Since there are very few animals per group, please provide the actual resource-equation calculation and explain how it supports n=2 per group. The manuscript should also clearly state that this sample size is not enough to reliably detect modest differences between groups.

**STATISTIC**

 The study included six animals, with (n=2 per group) in treatment group. Although the manuscript mentions using repeated-measures ANOVA or Friedman tests, as well as between-group ANOVA or Kruskal-Wallis tests, having only two animals per group makes it hard to draw reliable conclusions. The authors should explain their choice of statistical methods more clearly and consider providing the exact non-parametric P values when relevant. In the discussion, they should also state that the results are exploratory rather than confirmatory.

**RESULTS AND TABLE(S)**

**Results**

 The result is well written on point. The Results section lists NGAL concentrations as mg/mL, but the table uses ng/mL. Please check and use the same units throughout the manuscript.

**Table(s)**

 1. The tables are described as showing biomarker levels *'among groups*', but the statistical methods show that Friedman testing was used for comparisons over time within each group, and Kruskal-Wallis testing was used for comparisons between groups. Please clarify in each table whether the P value refers to within-group comparison over time, between-group comparison, or another type of analysis. Also, add clear footnotes to each table to help readers understand the significance of the analysis.

 2. Table 4 shows P=0.000 for creatinine in Groups 1 and 2, even though the median creatinine stays at 1.50 mg/dL at T1, T2, and T3. This result does not seem to match the descriptive statistics or the use of the Friedman test. Please check the individual-level raw data and confirm the analysis, including the test used, the test statistic, and the exact P value. Also, make sure to clearly separate between-group and within-group comparisons.

**Discussion**

 1. The manuscript uses terms like 'early norepinephrine group', 'norepinephrine-only group', and 'early norepinephrine with maintenance fluid group' interchangeably. Please select one standard term and use it consistently throughout the manuscripts and tables.

 2. The manuscript refers to cystatin C as a "structural biomarker" but also as an indicator of glomerular filtration. Please ensure that the classification of both NGAL and cystatin C is scientifically consistent throughout the manuscript.

 3. Some minor language edits are needed. For example, the phrase "Further studies with larger cohorts are warranted to validate these findings and improve statistical" is incomplete. Also, please revise the wording of "predominant cause" and "early detection of renal injury."

**Conclusion**

 The authors state that NGAL and cystatin C showed earlier changes than serum creatinine, but the timing differences were not statistically significant. It would be more accurate to describe these as "suggestive temporal patterns" instead of claiming that NGAL and cystatin C offer better early detection of AKI. Please revise this section so that the results match the findings and reflect the statistical analysis.

**Reference**

 Clear, well-formatted, and focused. However, please use more recent references.

Is the work clearly and accurately presented and does it cite the current literature?

Partly

If applicable, is the statistical analysis and its interpretation appropriate?

No

Are all the source data underlying the results available to ensure full reproducibility?

Yes

Is the study design appropriate and is the work technically sound?

Partly

Are the conclusions drawn adequately supported by the results?

No

Are sufficient details of methods and analysis provided to allow replication by others?

Yes

Reviewer Expertise:

Heart failure, cardiorenal syndrome, and cardiovascular epidemiology,

We confirm that we have read this submission and believe that we have an appropriate level of expertise to confirm that it is of an acceptable scientific standard, however we have significant reservations, as outlined above.

# Response

**Published:** 2026-10-05

**Authors:** Andriamuri Primaputra

**Affiliations:** Lubis, Indonesia

Dear Reviewer,

 We sincerely thank the Reviewer for the careful and constructive assessment of our manuscript. We agree that the original version did not sufficiently distinguish exploratory temporal biomarker observations from statistically supported evidence of acute kidney injury (AKI). We have therefore substantially revised the manuscript to improve methodological transparency, statistical interpretation, terminological consistency, and alignment between the study findings and its conclusions. Because this study included only six animals (n=2 per group), we have deliberately avoided introducing post hoc analyses that would imply a level of inferential certainty not supported by the experimental design.

 Below, we respond point-by-point. Reviewer comments are reproduced in bold, followed by our responses and the corresponding changes made in the revised manuscript.

**1. Reviewer comment**

**Title and overall framing: The title is acceptable, although other reviewers have suggested a title revision.**

**Response: **We appreciate this suggestion and agree that the original title could imply that the study demonstrated early diagnostic identification of sepsis-associated AKI. Given the very small sample size and the absence of statistically significant temporal changes, we revised the title to reflect the exploratory nature of the work and its actual analytical focus. The revised title emphasizes temporal biomarker dynamics during septic shock rather than diagnostic superiority.

**Revision made: **Title revised to: “Temporal Dynamics of NGAL, Cystatin C, Bladder Volume, and Serum Creatinine During Septic Shock in a Porcine (Sus scrofa) Model: An Exploratory Comparison of Fluid and Vasopressor Strategies.”

**2. Reviewer comment**

**Abstract: The novelty/knowledge gap is insufficiently stated. Results should be statistically supported, and the conclusion should clearly state the key findings in context.**

**Response: **We agree. The Abstract has been rewritten so that the Background explicitly identifies the knowledge gap as the limited characterization of the temporal behavior of renal biomarkers during septic shock under different resuscitation strategies. The Results now report the observed numerical fluctuations without interpreting them as statistically demonstrated early AKI. All reported inferential findings are accompanied by their statistical context, and the Conclusion now explicitly states that none of the biomarker changes reached statistical significance and that the study did not establish KDIGO-defined AKI.

**Revision made: **Abstract revised to distinguish descriptive temporal patterns from statistically supported findings and to present the study as an exploratory pilot investigation.

**3. Reviewer comment**

**Introduction: The Introduction is well written, but the knowledge gap and novelty should be made clearer using previous evidence.**

**Response: **We agree. The final portion of the Introduction has been revised to clarify that NGAL and cystatin C have been investigated as potentially earlier markers of renal dysfunction, but that their temporal behavior during septic shock may be influenced by the timing and type of hemodynamic resuscitation. The present study is therefore framed as an exploratory comparison of temporal biomarker dynamics rather than a diagnostic accuracy study.

**Revision made: **Knowledge-gap and study-rationale paragraphs revised; the previous hypothesis claiming superior or earlier AKI detection has been replaced by a hypothesis-generating temporal-dynamics framework.

**4. Reviewer comment**

**Methods—AKI definition: Please clarify how AKI was defined. Was a predefined diagnostic criterion used, or were biomarker changes interpreted as AKI?**

**Response: **We agree that this distinction is essential. The study did not use a predefined KDIGO-based categorical AKI endpoint. Serum creatinine was measured longitudinally, while bladder volume was assessed by ultrasound as an indirect measure of urinary accumulation. Because continuous urine output was not collected and normalized to body weight and time, a KDIGO urine-output criterion could not be applied. Accordingly, the revised manuscript explicitly states that biomarker fluctuations were evaluated as temporal renal responses and were not considered sufficient, by themselves, to establish AKI.

**Revision made: **An explicit subsection describing the definition and assessment of AKI has been added. The Results, Discussion, and Conclusion have also been revised to avoid equating biomarker fluctuations with established AKI.

**5. Reviewer comment**

**Methods—bladder volume methodology: Please provide details regarding the ultrasound procedure, timing, interpretation, limitations, and whether the three groups included a true control group.**

**Response: **We agree. The revised Methods now specify that bladder volume was assessed at three predefined experimental time points: T1 after hemodynamic stabilization and before septic shock induction, T2 during established septic shock, and T3 following the assigned resuscitation intervention. Ultrasound was used to estimate the volume of urine present in the bladder at each time point, representing urinary accumulation rather than urine production over a standardized time interval. At the end of the protocol, after euthanasia, the accumulated urine was completely collected from the bladder and directly measured in milliliters. Because urine was not continuously collected and was not normalized to body weight per hour, these measurements cannot be expressed as mL/kg/h and were not used to establish a KDIGO urine-output criterion. We also clarified that there was no non-septic control group: all animals underwent the same septic shock induction protocol, and the three randomized groups represented different resuscitation strategies.

**Revision made: **Methods rewritten for bladder-volume assessment and study-group structure. Table 3 has been renamed to identify the variable as ultrasonographically assessed bladder volume rather than conventional urine output.

**6. Reviewer comment**

**Sample size: Please provide the actual resource-equation calculation and explain how n=2/group was supported. Please acknowledge that this is insufficient to detect modest differences.**

**Response: **We agree that the sample size is the principal limitation of the study. The experiment included six animals distributed equally across three resuscitation strategies (n=2/group). The study was designed as an exploratory pilot experiment intended to characterize feasibility and temporal patterns rather than to provide definitive hypothesis testing. With six animals and three experimental groups, the available residual/error degrees of freedom are extremely limited. We therefore do not claim that the sample size provides adequate power to detect modest between-group differences. The revised manuscript explicitly states that the study is hypothesis-generating and that larger samples are required for confirmatory inference.

**Revision made: **The Sample Size and Limitations sections have been revised to explicitly describe the exploratory rationale and the inability of n=2/group to reliably detect modest treatment effects.

**7. Reviewer comment**

**Statistics: Given n=2/group, reliable inference is difficult. Please explain the choice of methods, provide exact non-parametric P-values, and clearly describe the analysis as exploratory.**

**Response: **We agree. The statistical analysis has been clarified to emphasize the repeated measurements within individual animals and the very small number of independent experimental units. Temporal changes within each group were evaluated using the Friedman test where appropriate for the non-parametric repeated-measures structure. Between-group comparisons, where considered, were treated as exploratory because the independent sample size was only two animals per group. P-values are presented as exact values where available from the current analysis. We have also revised the manuscript to make clear that statistical testing is hypothesis-generating and should not be interpreted as confirmatory evidence.

**Revision made: **The Statistical Analysis section, Results, table footnotes, and Discussion have been revised to distinguish within-group temporal comparisons from between-group comparisons and to emphasize the exploratory nature of the analyses.

**8. Reviewer comment**

**Results/Table 1–4: NGAL units appear inconsistent (mg/mL versus ng/mL). Please standardize them.**

**Response: **We agree and thank the Reviewer for identifying this inconsistency. The NGAL unit has been standardized throughout the manuscript according to the assay/table reporting as ng/mL. The inconsistent mg/mL terminology has been removed.

**Revision made: **NGAL units standardized throughout the Abstract, Methods, Results, Tables, figure labels where applicable, and Discussion.

**9. Reviewer comment**

**Tables: The tables are described as comparisons among groups, but the P-values appear to represent Friedman tests within groups over time. Please clarify and separate within-group from between-group comparisons.**

**Response: **We agree. The table titles and footnotes have been revised so that they explicitly state that the reported P-values represent within-group temporal comparisons across T1, T2, and T3 using the Friedman test. They are not presented as between-group P-values. We have also revised the Results narrative to avoid describing these values as evidence of differences between the three resuscitation strategies.

**Revision made: **Tables 1–4 have been retitled to emphasize temporal changes within each resuscitation group, and footnotes now specify the statistical test and comparison represented by each P-value.

**10. Reviewer comment**

**Table 4: The previous P=0.000 issue should be checked against the raw data, including the test statistic and exact P-value, and within- versus between-group comparisons should be separated.**

**Response: **We agree. The serum creatinine analysis was rechecked in the current dataset and the previously reported P=0.000 values have been corrected. The current within-group Friedman P-values are 0.156 for the Lactated Ringer’s group, 0.135 for the Lactated Ringer’s plus early norepinephrine group, and 0.156 for the early norepinephrine plus maintenance fluid group. These values are interpreted strictly as within-group temporal comparisons. No claim of statistically significant between-group difference is made.

**Revision made: **Table 4 and the corresponding Results paragraph have been corrected and aligned with the current statistical output. The table footnote now clearly identifies the comparison as within-group over time.

**11. Reviewer comment**

**Discussion: Please standardize the group names, clarify the meaning of cystatin C, and correct wording such as 'structural biomarker,' 'predominant cause,' 'early detection of renal injury,' and the incomplete phrase 'improve statistical.'**

**Response: **We agree with all of these editorial and scientific corrections. Group terminology has been standardized throughout the manuscript. Cystatin C is now described primarily as a marker related to glomerular filtration/function rather than as a structural biomarker. The language regarding renal injury has been revised to describe temporal renal biomarker dynamics rather than definitive early detection. The phrase 'predominant cause' has been replaced with more appropriate wording, and the incomplete phrase 'improve statistical' has been corrected to 'improve statistical power.'

**Revision made: **Terminology and wording were systematically revised throughout the Methods, Results, Discussion, and Conclusion.

**12. Reviewer comment**

**Discussion: The manuscript should better address the potential renal effects of norepinephrine, including renal microcirculation, despite restoration of systemic MAP.**

**Response: **We agree and have expanded the Discussion. Restoration of systemic MAP does not necessarily imply normalization of intrarenal microcirculatory perfusion. Norepinephrine may improve renal perfusion pressure through systemic hemodynamic stabilization, while its vasoconstrictive effects may also influence regional renal vascular distribution. In this pilot experiment, the observed temporal behavior of cystatin C in the early norepinephrine plus maintenance-fluid group should therefore be regarded only as a hypothesis-generating observation. Given n=2/group and the absence of statistically significant differences, the present data cannot determine whether the observed pattern reflects a true pharmacologic effect, inter-animal variability, or other experimental factors.

**Revision made: **Discussion expanded to provide a balanced mechanistic interpretation without attributing causality to norepinephrine.

**13. Reviewer comment**

**Conclusion: Please replace the claim of earlier changes with 'suggestive temporal patterns' and ensure that the conclusion matches the statistical analysis.**

**Response: **We fully agree. The Conclusion has been revised to state that the study demonstrated suggestive temporal patterns in NGAL and cystatin C during septic shock under different resuscitation strategies, but that none of the observed biomarker changes reached statistical significance. We also explicitly state that the study did not establish KDIGO-defined AKI because standardized urine-output measurements were unavailable and serum creatinine did not demonstrate statistically significant temporal changes. The conclusion now presents the findings as preliminary evidence requiring validation rather than as proof of earlier AKI detection.

**Revision made: **Conclusion completely revised to align with the statistical results and exploratory study design.

**14. Reviewer comment**

**References: Please add more recent references.**

**Response: **We agree. The reference list has been updated to incorporate more recent literature addressing sepsis-associated AKI, renal biomarkers including NGAL and cystatin C, renal microcirculation, and contemporary approaches to sepsis and AKI assessment, while retaining key foundational studies necessary for the biological rationale.

**Revision made: **Recent literature has been incorporated into the revised Introduction and Discussion to place the pilot findings in the context of contemporary evidence.

 Additional manuscript-wide revisions

 In addition to the point-by-point revisions above, we performed a manuscript-wide consistency review. We corrected several editing artifacts in the Methods section, standardized terminology and capitalization for NGAL and cystatin C, clarified the distinction between bladder volume and conventional urine output, and ensured that statements regarding AKI are consistent with the actual measurements obtained. We also revised the Discussion to emphasize the exploratory and hypothesis-generating nature of the study.

 Closing response

 We are grateful to Reviewer 3 for the detailed comments. The revisions have substantially improved the methodological transparency and scientific interpretation of the manuscript. Most importantly, we have reframed the study from a claim of early AKI identification to an exploratory assessment of temporal renal biomarker dynamics during septic shock. We believe this revised framing more accurately reflects the data and appropriately communicates both the potential relevance and the limitations of this pilot experiment.

 Sincerely,

 The Authors

# Reviewer report: Reviewer response for version 1

**DOI:** 10.5256/f1000research.201765.r500985

**Published:** 2026-08-26

**Authors:** Abeer Cheaid Yousif Al-Fatlawi

**Affiliations:** 1 Pathological analysis, University of Kerbala, Kerbala, Kerbala, Iraq

Review Points for the manuscript title  

 " **Early identification of sepsis-induced acute kidney injury utilizing NGAL, Cystatin C, urine output, and serum creatinine in a porcine ( *Sus scrofa*) model of septic shock: A comparative study of fluid and vasopressor strategies** "

 I have thoroughly reviewed the provided manuscript, and here are the detailed feedback and areas for improvement:

 1. Title

 Current Title:

**Early identification of sepsis-induced acute kidney injury utilizing NGAL, Cystatin C, urine output, and serum creatinine in a porcine ( *Sus scrofa*) model of septic shock: A comparative study of fluid and vasopressor strategies**

 According to the nature of the manuscript as (an exploratory preclinical study) and the very small sample size used, I suggest the following title:

**Exploratory Evaluation of NGAL and Cystatin C for Early Detection of Sepsis Associated with Acute Kidney Injury in Sus scrofa: A Pilot Experimental Study in Pigs**

**2- Methodology**

- Since only six animals were used in the study, two in each group, this will greatly weaken the statistical power to detect significant differences between the groups. Therefore, either the sample size should be increased, or the title, objectives, and methodology should be reformulated to be a pilot exploratory study.
- A urinary catheter must be used for direct and accurate measurement of urine volume in (ml/kg/hr) for precise scientific comparison in the measurement of biomarkers.
- The monitoring period should be extended to 48 hours or more because functional changes in the kidneys need time to appear and then for biomarkers to be released.
- Add correlation analysis between biomarkers to confirm the synchronous increase.

3- Results

- In Table 4, for creatinine, I noticed several inconsistencies when the significance P value (0.000) appeared in groups where the values ​​did not change 1,50 (1,40–1,60). The statistical analysis should be reviewed and an explanation for this contradiction is needed if it does not change.
- Since the study examines the porcine model and structural biomarkers, it would be best to add a renal histopathological study to the manuscript to increase its scientific strength by microscopic evidence and demonstrate the structural damage in the renal tube and its relationship to elevated biomarkers.
- Add to the results correlation analysis of Cystatin C levels with mean arterial pressure (MAP) in the group of early norepinephrine to explain the delayed rise you found.
- Add Regression analysis (Linear Mixed Model LME) to assess the ability of biomarkers (NGAL or Cystatin C) to predict the occurrence of septic shock or changes in kidney function across the same animal at different time points (T1, T2, T3).
- Statistical note: In the discussion section, it should be noted that the correlation or regression results are exploratory and not generalizable due to the limited sample size.
- Make sure to write the units of measurement correctly; for example: state NGAL as mg/mL in the text and ng/ml in the tables.
- The limitations of the study must be stated clearly and in greater detail and should not be limited to the small sample size but should also include: the biological differences between humans and pigs, the effect of anesthesia prior to euthanasia on kidney structure, and the method of urine collection from the bladder pigs.
- The researcher should explain how incorporating biomarkers (Cystatin C and NGAL) into the study can change clinical management in ICU resuscitation protocols rather than simply stating their elevation.
- Interpretation of the effect of norepinephrine and how the vasoconstrictors can affect internal microcirculation in the kidney, even with stable systemic blood pressure (when the group that received only norepinephrine showed a delayed increase in Cystatin C).
- In general, the study is potentially valuable, but the methodology and limitations really require clarification and revision before it can be considered scientifically robust. Decision: approved with reservation 

Is the work clearly and accurately presented and does it cite the current literature?

Partly

If applicable, is the statistical analysis and its interpretation appropriate?

No

Are all the source data underlying the results available to ensure full reproducibility?

Yes

Is the study design appropriate and is the work technically sound?

Partly

Are the conclusions drawn adequately supported by the results?

Partly

Are sufficient details of methods and analysis provided to allow replication by others?

Partly

Reviewer Expertise:

Human and renal physiology,  clinical biochemistry

I confirm that I have read this submission and believe that I have an appropriate level of expertise to confirm that it is of an acceptable scientific standard, however I have significant reservations, as outlined above.

# Response

**Published:** 2026-10-05

**Authors:** Andriamuri Primaputra

**Affiliations:** Lubis, Indonesia

Dear Reviewer,

 We sincerely thank the reviewer for the careful evaluation of our manuscript and for the constructive recommendations. We appreciate the reviewer’s comments regarding the exploratory nature of the study, urine-output methodology, duration of observation, biomarker relationships, statistical analysis, renal histopathology, biomarker units, limitations, and the potential clinical implications of NGAL and cystatin C.

 We have carefully reconsidered the manuscript in light of these comments. Because this study included only six experimental animals (n = 2 per group), we have deliberately avoided introducing statistical analyses that would create a false impression of confirmatory power. Where additional analyses are supported by the available individual-level data, they should be presented as exploratory and hypothesis-generating. Where the requested measurement was not performed in the original experiment, we have clarified the limitation rather than retrospectively inferring or generating data.

 The point-by-point responses are provided below.

**Reviewer Comment**

 1. Title. Given the exploratory preclinical nature of the manuscript and the very small sample size, the reviewer suggests the title: “Exploratory Evaluation of NGAL and Cystatin C for Early Detection of Sepsis Associated with Acute Kidney Injury in Sus scrofa: A Pilot Experimental Study in Pigs.”

**Response**

 We thank the reviewer for this recommendation and agree that the title should clearly reflect the exploratory pilot nature of the study. However, because the present study did not establish KDIGO-defined acute kidney injury and the changes in NGAL and cystatin C were not statistically significant, we believe that the phrase “early detection of sepsis-associated acute kidney injury” remains stronger than supported by the data. We have therefore adopted a more conservative title that describes the actual study objective and findings without implying diagnostic confirmation: “Temporal Dynamics of NGAL, Cystatin C, Bladder Volume, and Serum Creatinine During Septic Shock in a Porcine (Sus scrofa) Model: An Exploratory Comparison of Fluid and Vasopressor Strategies.” This revised title emphasizes biomarker trajectories and the exploratory design.

**Reviewer Comment**

 2. Methodology: Since only six animals were used, statistical power is greatly limited. Therefore, either the sample size should be increased, or the title, objectives, and methodology should be reformulated as a pilot exploratory study.

**Response**

 We agree with the reviewer. Increasing the number of animals retrospectively is not possible because the experimental procedures have already been completed and the study was designed in accordance with the reduction principle for animal research. We have therefore consistently reframed the study as an exploratory pilot study. The Methods, objectives, Results, Discussion, and Conclusion now explicitly state that the study is hypothesis-generating and that treatment-group comparisons are exploratory rather than confirmatory. We also emphasize individual animal trajectories and the need for larger adequately powered studies to validate the observations.

**Reviewer Comment**

 3. Methodology: A urinary catheter must be used for direct and accurate measurement of urine volume in mL/kg/h.

**Response**

 We agree that continuous catheter-based urine collection would provide a more rigorous assessment of urine output and would allow direct application of KDIGO urine-output criteria. However, urinary catheterization was not performed in the original experiment, and urine output in mL/kg/h therefore cannot be retrospectively calculated from the available measurements. We have clarified this limitation in the Methods and Discussion. We have also revised Table 3 so that the measured variable is described as ultrasonographically assessed bladder volume rather than urine output. We explicitly state that these measurements cannot be interpreted as standardized urine output or used to establish KDIGO-defined AKI. We agree that future studies should incorporate continuous urinary catheterization with weight- and time-normalized urine-output measurement.

**Reviewer Comment**

 4. Methodology: The monitoring period should be extended to 48 hours or more because functional renal changes need time to appear and biomarkers may be released later.

**Response**

 We appreciate this recommendation. We agree that a longer observation period would allow assessment of delayed creatinine changes and more complete biomarker kinetics. The current protocol was limited to the predefined experimental observation period and therefore cannot be retrospectively extended. We have added the limited observation period as an explicit limitation and have revised the Discussion to state that the study captures early temporal responses rather than the complete evolution of septic AKI. We also identify extended follow-up (at least 48 hours where ethically and experimentally feasible) as an important component of future validation studies.

**Reviewer Comment**

 5. Methodology: Add correlation analysis between biomarkers to confirm the synchronous increase.

**Response**

 We agree that biomarker correlation could provide useful exploratory information. However, the present manuscript does not establish a synchronous increase: NGAL and cystatin C demonstrated different numerical trajectories across groups, and the temporal changes were not statistically significant. Accordingly, we have avoided describing correlation as confirmation of synchronous biomarker elevation. If individual-level paired measurements are used for an exploratory correlation analysis, the result will be explicitly labeled exploratory and interpreted cautiously because only six animals contributed repeated measurements. We will not use such an analysis to claim diagnostic validity or generalizability.

**Reviewer Comment**

 6. Results: In Table 4, the P value of 0.000 appears inconsistent with unchanged creatinine values. The statistical analysis should be reviewed and an explanation provided.

**Response**

 We thank the reviewer for identifying this issue. We re-checked the underlying creatinine data and repeated the within-group analysis. The previously reported P = 0.000 values were incorrect and have been corrected. The revised Table 4 reports P = 0.156 for Group 1, P = 0.135 for Group 2, and P = 0.156 for Group 3 using the Friedman test. The Results section has been revised accordingly. We apologize for this statistical reporting error.

**Reviewer Comment**

 7. Results: Because the study examines a porcine model and structural biomarkers, renal histopathology should be added to demonstrate tubular structural damage and its relationship with elevated biomarkers.

**Response**

 We agree that renal histopathology would substantially strengthen a mechanistic assessment of tubular injury. However, renal histopathological examination was not performed as part of the original experimental protocol, and therefore histological findings cannot be added retrospectively without new experimental material. Importantly, we have also corrected the terminology in the manuscript: NGAL is described as a biomarker associated with renal tubular injury/stress, whereas cystatin C primarily reflects glomerular filtration rather than structural tubular injury. We have explicitly acknowledged the absence of renal histopathology as a limitation and have identified histopathological assessment as an important endpoint for future studies designed to validate the relationship between biomarker trajectories and structural renal injury.

**Reviewer Comment**

 8. Results: Add correlation analysis of cystatin C with mean arterial pressure (MAP) in the early norepinephrine group to explain the delayed rise.

**Response**

 We appreciate this suggestion. The observed delayed increase in cystatin C in the early norepinephrine group is biologically interesting; however, the current manuscript does not provide sufficient individual-level MAP data in the Results to establish a reliable correlation. With only two animals in the early norepinephrine group, a formal correlation coefficient would also be extremely unstable and would not provide meaningful inferential evidence. We have therefore strengthened the descriptive discussion of the temporal relationship between hemodynamic support and cystatin C while explicitly stating that the proposed relationship between MAP and cystatin C remains hypothesis-generating. We have not presented a formal MAP–cystatin C correlation as confirmatory evidence because the available sample size is insufficient.

**Reviewer Comment**

 9. Results: Add regression analysis (Linear Mixed Model, LME) to assess whether NGAL or cystatin C predicts septic shock or changes in kidney function across T1, T2, and T3.

**Response**

 We appreciate the suggestion to explore longitudinal relationships. However, a linear mixed-effects model intended to establish biomarker predictive ability would be inappropriate as a confirmatory analysis in this dataset because there are only six experimental units and two animals per treatment group. Repeated measurements within an animal do not create independent experimental units, and fitting a complex predictive model with this number of animals would risk severe overfitting and unstable estimates. In addition, the present study did not have a predefined predictive endpoint for biomarker-based prediction of septic shock or KDIGO-defined AKI. We have therefore not added an LME model as a confirmatory predictive analysis. Instead, the revised manuscript explicitly states that the temporal analyses are exploratory and that larger studies with adequate numbers of independent animals are required before longitudinal predictive modeling can be meaningfully performed.

**Reviewer Comment**

 10. Statistical note: Correlation or regression results should be described as exploratory and not generalizable due to the limited sample size.

**Response**

 We fully agree. The revised manuscript emphasizes that any exploratory association or temporal analysis is hypothesis-generating and cannot be generalized to other animals, treatment strategies, or human patients. We have strengthened the statistical limitations in the Discussion and state that larger, adequately powered studies are required for validation.

**Reviewer Comment**

 11. Ensure units are written correctly; for example, NGAL is stated as mg/mL in the text and ng/mL in the tables.

**Response**

 We agree and have standardized the units throughout the manuscript. NGAL and cystatin C concentrations are now reported using the same unit notation in the Abstract, Methods, Results, tables, and Discussion. We also corrected inconsistent capitalization and formatting of units so that the text and tables are internally consistent.

**Reviewer Comment**

 12. Limitations should include not only sample size but also biological differences between humans and pigs, effects of anesthesia prior to euthanasia on kidney structure, and the method of urine collection from the bladder.

**Response**

 We agree and have substantially expanded the Limitations discussion. The revised manuscript now addresses: (1) the very small number of animals and the resulting limited statistical power; (2) biological and physiological differences between pigs and humans that may limit clinical translation; (3) the limited observation period; (4) the absence of standardized catheter-based urine-output measurements; (5) the absence of renal histopathology; and (6) the fact that terminal bladder urine collection represents accumulated urine volume rather than standardized urine production in mL/kg/h. We also clarify that euthanasia was performed under deep general anesthesia and in accordance with the approved animal protocol. Because renal histopathology was not performed, we do not make claims about microscopic structural injury. The potential influence of anesthesia and terminal experimental conditions on renal physiology is acknowledged as a limitation when interpreting any future structural assessment.

**Reviewer Comment**

 13. The researcher should explain how incorporating NGAL and cystatin C into ICU resuscitation protocols could change clinical management rather than simply stating their elevation.

**Response**

 We appreciate this clinically important point. We have revised the Discussion to avoid suggesting that biomarker elevation alone should alter ICU management. Instead, we describe a potential future role for these biomarkers as adjuncts to conventional clinical assessment. In a validated clinical setting, earlier evidence of renal stress or injury could potentially prompt closer renal monitoring, more careful assessment of fluid balance, avoidance of unnecessary fluid accumulation or nephrotoxic exposure, and reassessment of hemodynamic targets. However, because the present animal study did not establish diagnostic accuracy, treatment benefit, or biomarker-guided outcome improvement, we explicitly state that these findings do not justify changing current ICU resuscitation protocols. Prospective clinical validation would be required before biomarker-guided management could be recommended.

**Reviewer Comment**

 14. Interpretation of norepinephrine: discuss how vasoconstrictors may affect renal microcirculation even with stable systemic blood pressure, particularly the delayed cystatin C increase in the norepinephrine-only group.

**Response**

 We agree and have expanded the Discussion of this observation. The revised manuscript explains that restoration of systemic MAP with norepinephrine does not necessarily imply normalization of intrarenal microcirculatory perfusion. Norepinephrine may restore vascular tone and systemic perfusion pressure while potentially altering intrarenal vascular distribution and microcirculatory dynamics. The delayed increase in cystatin C observed in the norepinephrine-only group is therefore discussed as a possible temporal renal response associated with the hemodynamic effects of vasopressor therapy. Importantly, because the study has only two animals in this group and the cystatin C change was not statistically significant, we explicitly state that this mechanism is speculative and hypothesis-generating rather than demonstrated by the present experiment.

 Additional Revisions Made in Response to Reviewer 

- The manuscript has been consistently reframed as an exploratory pilot study.
- The title and study objective have been revised to avoid implying definitive diagnosis of AKI.
- Table 3 terminology has been changed from “Urine Output” to ultrasonographically assessed bladder volume.
- The Methods now clarify that bladder-volume measurements cannot be converted into standardized urine output in mL/kg/h.
- The Discussion now explicitly states that KDIGO-defined AKI was not established.
- The distinction between NGAL as a tubular injury/stress-associated biomarker and cystatin C as a filtration marker has been clarified.
- The limitations section has been expanded to address translational differences, observation period, urine methodology, lack of histopathology, and sample size.
- The clinical implications of biomarker use have been reframed as a potential future adjunct rather than a basis for changing current ICU protocols.
- The interpretation of norepinephrine and potential intrarenal microcirculatory effects has been expanded while emphasizing that the present finding is hypothesis-generating.

We sincerely thank Reviewer for these constructive recommendations. We believe that the revisions improve the methodological transparency and scientific rigor of the manuscript while ensuring that the conclusions remain proportionate to the available experimental evidence.

 Sincerely,

 The Authors

# Reviewer report: Reviewer response for version 1

**DOI:** 10.5256/f1000research.201765.r500979

**Published:** 2026-08-20

**Authors:** Rohini Saha

**Affiliations:** 1 All India Institute of Medical Sciences, New Delhi, Delhi, India

The article "Early identification of sepsis-induced acute kidney injury utilizing NGAL, Cystatin C, urine output, and serum creatinine in a porcine ( *Sus scrofa*) model of septic shock: A comparative study of fluid and vasopressor strategies" is a good attempt to study utility of NGAL and Cys C in prediction of AKI in sepsis. The development and use of a porcine model is good. The authors have followed the ARRIVE 2.0 recommendations.

 However, the manuscript in the current form has several limitations that substantially affect the strength of its conclusions.

 1.The authors conclude that NGAL and cystatin C showed earlier changes than serum creatinine. However, the reported changes in NGAL and cystatin C are not statistically significant in any of the groups (Table 1 and Table 2). Therefore, the statement that these biomarkers provide “earlier identification” of AKI should be toned down. The authors should clearly distinguish between an observed numerical change and a statistically supported biomarker response.

 2. Creatinine values remain exactly the same at T1, T2 and T3 in Groups 1 and 2 (1.50 mg/dL and 1.45 mg/dL, respectively), yet the reported P value is 0.000. This appears inconsistent with the data presented in the table. The authors should re-check the statistical analysis and correct the P values if there is an error.

 3. 

1. Serum creatinine does not increase, and the reported urine measurements are not expressed according to standard urine-output criteria (mL/kg/h). Therefore, it is difficult to establish that the animals actually developed AKI. The authors should clarify whether the study is demonstrating AKI or simply early changes in renal biomarkers during septic shock.
2. The urine-output measurement needs clarification.
  The Methods state that urine output was indirectly assessed by ultrasound bladder-volume measurements and that urine production was not calculated in mL/kg/h. However, Table 3 reports the outcome as “Urine Output (ml).” Please clarify how bladder volume was converted into urine output and whether the bladder was emptied between measurements.
3. The sample size is extremely small.
  Only six animals were used, with two animals per treatment group. Although the authors acknowledge this limitation, the manuscript should be more cautious when comparing the three treatment strategies. The study would be better presented as an exploratory/pilot study, with greater emphasis on individual animal data and biomarker trajectories rather than statistical comparisons.

Minor comments

1. The authors refer to both NGAL and cystatin C as “structural biomarkers.” This terminology should be clarified, as cystatin C primarily reflects renal filtration rather than structural tubular injury.

Overall recommendation

**Major revision.** The study is potentially interesting, but the statistical results and interpretation need to be carefully revised. In particular, the reported P=0.000 for unchanged creatinine values should be checked, and the conclusions regarding the early diagnostic value of NGAL and cystatin C should be appropriately toned down given the lack of statistically significant changes.

Is the work clearly and accurately presented and does it cite the current literature?

Yes

If applicable, is the statistical analysis and its interpretation appropriate?

No

Are all the source data underlying the results available to ensure full reproducibility?

No

Is the study design appropriate and is the work technically sound?

No

Are the conclusions drawn adequately supported by the results?

No

Are sufficient details of methods and analysis provided to allow replication by others?

No

Reviewer Expertise:

Liver biology, Renal Biology, Biomarkers, ACLF, Immunology, Neutrophil

I confirm that I have read this submission and believe that I have an appropriate level of expertise to state that I do not consider it to be of an acceptable scientific standard, for reasons outlined above.

# Response

**Published:** 2026-10-05

**Authors:** Andriamuri Primaputra

**Affiliations:** Lubis, Indonesia

Dear Reviewer,

 We sincerely thank you for your careful and constructive review of our manuscript. We greatly appreciate your comments regarding the interpretation of the renal biomarkers, the statistical analysis, urine-output methodology, sample size, and biomarker terminology. We have carefully re-examined the raw data, statistical analysis, and manuscript text in response to your comments. The manuscript has been substantially revised to ensure that the conclusions are appropriately aligned with the exploratory nature of the study and the limitations of the experimental design.

 In particular, we have toned down the interpretation of NGAL and cystatin C, clarified that the study does not establish KDIGO-defined acute kidney injury, corrected the creatinine statistical results, clarified the bladder-volume methodology, strengthened the pilot-study framing, and revised the terminology used to describe NGAL and cystatin C.

 Below, we provide a point-by-point response to each comment. Changes in the revised manuscript are indicated in the corresponding sections.

**Reviewer Comment**

 1. The authors conclude that NGAL and cystatin C showed earlier changes than serum creatinine. However, the reported changes in NGAL and cystatin C are not statistically significant in any of the groups (Table 1 and Table 2). Therefore, the statement that these biomarkers provide “earlier identification” of AKI should be toned down. The authors should clearly distinguish between an observed numerical change and a statistically supported biomarker response.

**Response**

 We thank the reviewer for this important observation and agree that the original wording could overstate the findings. We re-examined the results and confirmed that the temporal changes in cystatin C and NGAL did not reach statistical significance in any treatment group. Accordingly, we have revised the manuscript throughout to distinguish numerical temporal fluctuations from statistically supported biomarker responses. We no longer present NGAL or cystatin C as providing definitive earlier identification or diagnosis of acute kidney injury. Instead, the findings are described as exploratory observations of renal biomarker dynamics during septic shock.

 Specifically, the Abstract Results and Conclusion, the Introduction hypothesis, the Discussion, and the Conclusion have been revised to emphasize that the observed changes were numerical and hypothesis-generating. We also revised the interpretation of NGAL and cystatin C so that their observed temporal patterns are not interpreted as evidence of diagnostic superiority over serum creatinine.

**Reviewer Comment**

 2. Creatinine values remain exactly the same at T1, T2 and T3 in Groups 1 and 2 (1.50 mg/dL and 1.45 mg/dL, respectively), yet the reported P value is 0.000. This appears inconsistent with the data presented in the table. The authors should re-check the statistical analysis and correct the P values if there is an error.

**Response**

 We thank the reviewer for identifying this inconsistency. We carefully re-checked the underlying creatinine data and repeated the within-group analysis. The previously reported P = 0.000 values were incorrect and have been corrected in the revised manuscript. The current Table 4 reports P = 0.156 for Group 1, P = 0.135 for Group 2, and P = 0.156 for Group 3 using the Friedman test. The Results section has also been revised to reflect the corrected analysis. We apologize for this statistical reporting error and appreciate the reviewer for bringing it to our attention.

**Reviewer Comment**

 3.1. Serum creatinine does not increase, and the reported urine measurements are not expressed according to standard urine-output criteria (mL/kg/h). Therefore, it is difficult to establish that the animals actually developed AKI. The authors should clarify whether the study is demonstrating AKI or simply early changes in renal biomarkers during septic shock.

**Response**

 We agree with the reviewer. The study should not be interpreted as demonstrating KDIGO-defined acute kidney injury. Serum creatinine did not demonstrate statistically significant temporal changes, and urine measurements were obtained by ultrasonographic assessment of bladder volume rather than continuous urine collection normalized to body weight and time. Therefore, the available data do not allow us to establish AKI according to standardized KDIGO creatinine or urine-output criteria.

 We have revised the manuscript to explicitly state that the study is an exploratory assessment of early renal biomarker dynamics during septic shock. The Discussion now clarifies that the observed NGAL and cystatin C changes may represent early renal responses or renal stress during septic shock rather than established AKI.

**Reviewer Comment**

 3.2. The urine-output measurement needs clarification. The Methods state that urine output was indirectly assessed by ultrasound bladder-volume measurements and that urine production was not calculated in mL/kg/h. However, Table 3 reports the outcome as “Urine Output (ml).” Please clarify how bladder volume was converted into urine output and whether the bladder was emptied between measurements.

**Response**

 We thank the reviewer for identifying this important methodological ambiguity. We have clarified that the ultrasonographic measurements represented bladder volume and were not converted into standardized urine output in mL/kg/h. Because urine was not continuously collected and the measured bladder volume did not represent urine production over a defined time interval, these measurements cannot be interpreted as KDIGO urine-output measurements.

 The Methods section has been revised to describe the ultrasonographic assessment as an indirect assessment of urinary accumulation. At the end of the experimental protocol, following euthanasia, the accumulated urine was completely collected from the bladder and directly measured in milliliters. Table 3 has also been revised so that the variable is reported as bladder volume rather than “Urine Output.” We have further clarified the interpretation of these measurements in the Results and Discussion sections.

 The statement concerning whether the bladder was emptied between serial measurements has been aligned with the actual experimental protocol and is now explicitly described in the revised Methods section.

**Reviewer Comment**

 3.3. The sample size is extremely small. Only six animals were used, with two animals per treatment group. Although the authors acknowledge this limitation, the manuscript should be more cautious when comparing the three treatment strategies. The study would be better presented as an exploratory/pilot study, with greater emphasis on individual animal data and biomarker trajectories rather than statistical comparisons.

**Response**

 We fully agree with the reviewer. The manuscript has been revised to consistently describe the study as an exploratory pilot study. The Methods section explicitly states that the study was designed to characterize temporal renal biomarker trajectories rather than provide confirmatory comparisons among treatment strategies. The statistical interpretation has also been strengthened: group-level comparisons are considered exploratory and hypothesis-generating rather than confirmatory.

 The Discussion has been revised to emphasize the very small number of experimental units (n = 2 per group), the potential influence of individual animal variability, and the inability to infer superiority of any resuscitation strategy from the present data. We have also strengthened the emphasis on individual temporal trajectories and recommend that larger studies incorporate individual-animal analyses and adequately powered comparisons.

**Reviewer Comment**

 Minor comment 1. The authors refer to both NGAL and cystatin C as “structural biomarkers.” This terminology should be clarified, as cystatin C primarily reflects renal filtration rather than structural tubular injury.

**Response**

 We thank the reviewer for this clarification and agree. We have removed the terminology that collectively classified NGAL and cystatin C as “structural biomarkers.” The revised manuscript distinguishes their biological roles: NGAL is described as a biomarker associated with renal tubular injury or stress, whereas cystatin C is described primarily as a marker of glomerular filtration and renal functional change. This terminology has been revised consistently in the Introduction and Discussion.

 Summary of Major Revisions

- The interpretation of NGAL and cystatin C has been toned down from early AKI identification to exploratory temporal biomarker dynamics.
- The creatinine statistical analysis was re-checked and the incorrect P values were corrected.
- The manuscript now explicitly states that KDIGO-defined AKI was not established in this experiment.
- The urine methodology has been clarified, and Table 3 has been revised to report bladder volume rather than urine output.
- The study is consistently framed as an exploratory pilot study because of the sample size of six animals (n = 2 per group).
- The terminology for NGAL and cystatin C has been revised to distinguish tubular injury/stress biomarkers from filtration/function markers.

We again thank the reviewer for the thoughtful and constructive comments. We believe that these revisions have substantially improved the scientific accuracy, transparency, and appropriate interpretation of the manuscript. We hope that the revised manuscript adequately addresses the concerns raised.

 Sincerely,

 The Authors
